# Supplementary material for: Age-related divergence of circulating immune responses in patients with solid tumors treated with immune checkpoint inhibitors
Source: Nat Commun. 2025 Apr 21;16:3531. doi: 10.1038/s41467-025-58512-z (PMC12012091; doi:10.1038/s41467-025-58512-z)
Supplement: Supplementary file 1 — Supplementary Information [file 41467_2025_58512_MOESM1_ESM.pdf]

Supplementary Tables and Supplementary Figures

Characteristics	Total patients (*n* = 104)	Age ≥65 (n=54)	Age <65 (n=50)
Cancer type - no. (%)			
Adrenal	1 (1.0)	0 (0.0)	1 (2.0)
Biliary Tract	2 (1.9)	1 (1.9)	1 (2.0)
Bladder	10 (9.6)	7 (13.0)	3 (6.0)
Breast	3 (2.9)	0 (0.0)	3 (6.0)
Cervical	2 (1.0)	0 (0.0)	2 (4.0)
Colorectal	1 (1.0)	1 (1.9)	0 (0.0)
Endometrial	2 (1.9)	2 (3.7)	0 (0.0)
Esophagogastric Junction	1 (1.0)	1 (1.9)	0 (0.0)
Head and Neck	9 (8.6)	2 (3.7)	7 (14.0)
Hepatocellular Carcinoma	30 (28.8)	18 (33.3)	12 (24.0)
Lung	1 (1.0)	0 (0.0)	1 (2.0)
Melanoma	3 (2.9)	1 (1.9)	2 (4.0)
Neuroendocrine	3 (2.9)	1 (1.9)	2 (4.0)
Pancreas	1 (1.0)	0 (0.0)	1 (2.0)
Renal Cell Carcinoma	24 (23.1)	13 (24.1)	11 (22.0)
Sarcoma	5 (4.8)	2 (3.7)	3 (6.0)
Squamous Cell Carcinoma of Skin	5 (4.8)	4 (7.4)	1 (2.0)
Vulvovaginal	1 (1.0)	1 (1.9)	0 (0.0)
irAE grade - no. (%)			
Grade 1	8 (7.7)	7 (13.0)	1 (2.0)
Grade 2	19 (18.3)	10 (18.5)	9 (18.0)
Grade 3	15 (14.4)	10 (18.5)	5 (10.0)
Grade 4	5 (4.8)	1 (1.9)	4 (8.0)
Grade 5	2 (1.9)	2 (3.7)	0 (0.0)
ICI treatment - no. (%)			
Anti-PD-1 or anti-PD-L1			
Atezolizumab	17 (16.3)	12 (22.2)	5 (10.0)
Cemiplimab	5 (4.8)	4 (7.4)	1 (2.0)
Nivolumab	14 (13.5)	7 (13.0)	7 (14.0)
Pembrolizumab	45 (43.3)	20 (37.0)	25 (50.0)
Anti-CTLA-4 and anti-PD-1			
Ipilimumab + Nivolumab	23 (22.1)	11 (20.4)	12 (24.0)
Best response - no. (%)[A]	(n = 87)	(n=48)	(n=39)
Complete response (CR)	9 (10.3)	7 (14.6)	2 (5.1)
Partial response (PR)	17 (19.5)	10 (20.8)	7 (17.9)
Stable disease (SD)	24 (27.6)	18 (37.5)	11 (28.2)
Progression	37 (42.5)	13 (27.1)	19 (48.7)

Supplementary Table 1: Expanded baseline characteristics in the total cohort

[A]Best response was only reported for patients with evaluable disease by Response Evaluation Criteria in Solid Tumors (RECIST). Abbreviations: irAE – immune related adverse event; ICI – immune checkpoint inhibitor; no. – number.

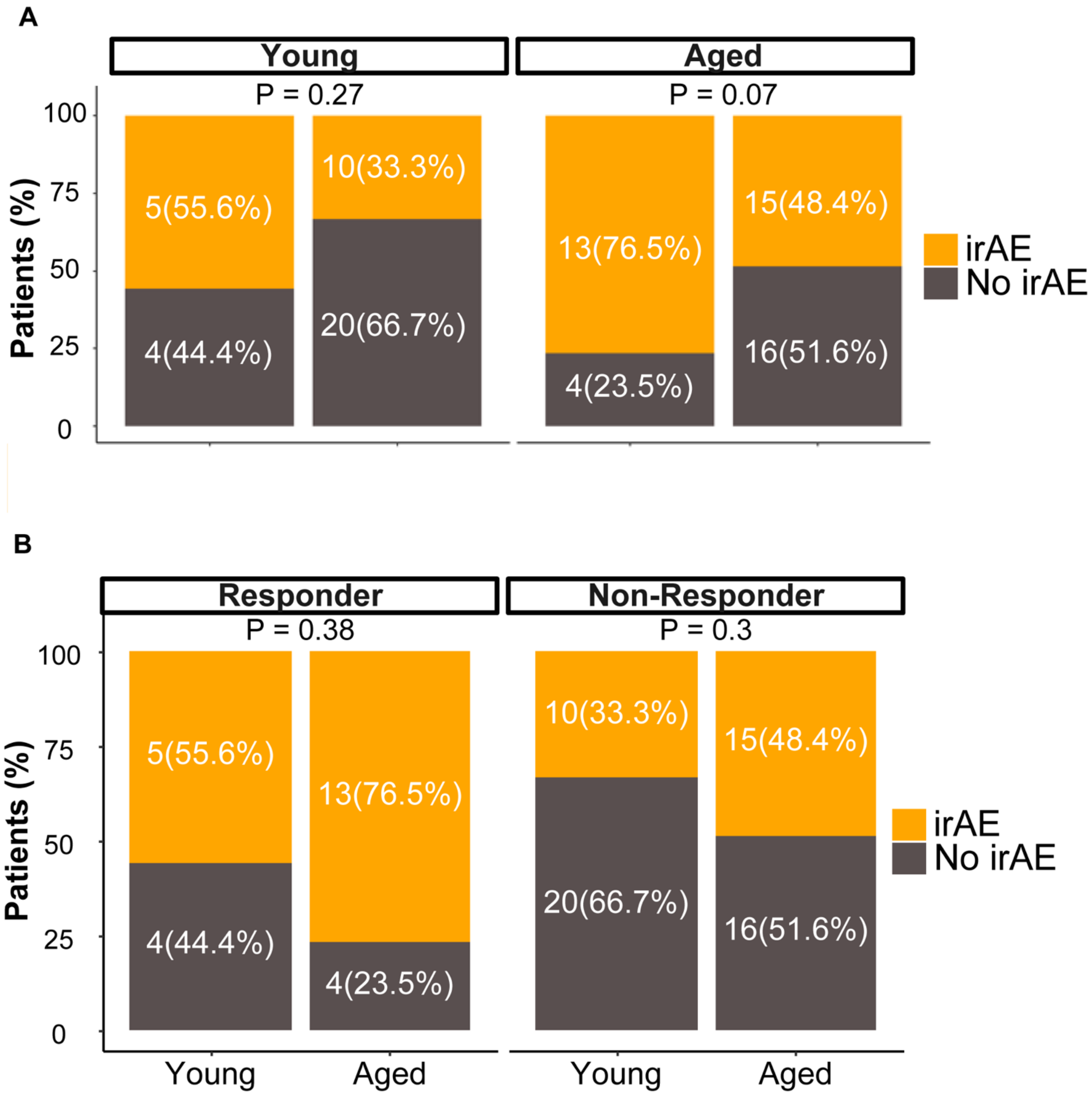

Supplementary Figure 1. Evaluation of immune related adverse events by patient immune checkpoint inhibitor response and age. (A) Proportion of patients in each age group who developed an immune related adverse event (irAE) divided by immune checkpoint inhibitor (ICI) response status. (B) Proportion of ICI responding and ICI non-responding patients who developed an irAE divided by patient age group. Statistical comparisons between quantitative measurements were performed using two-sided Wilcoxon rank-sum test and Fisher's exact test for categorical variables. Source data are provided as a Source Data file.

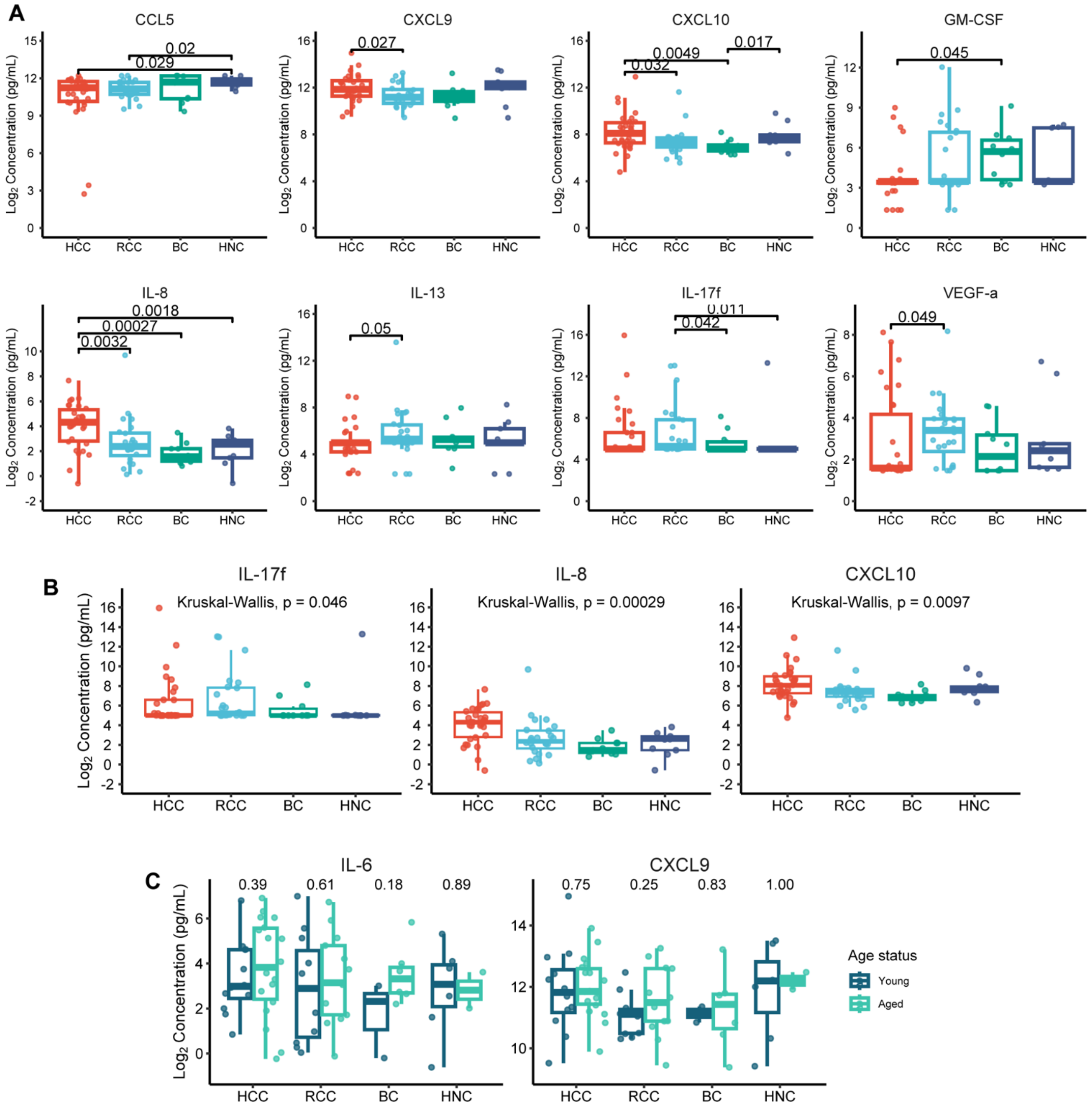

Supplementary Figure 2. Baseline cytokine expression by cancer type.
Group differences of baseline cytokine concentrations (n=104) were compared for the four most common cancer types: hepatocellular carcinoma (HCC, n=30), renal cell carcinoma (RCC, n=24), bladder cancer (BC, n=10), and head and neck (HNC, n=9). **(A)** Log_2 transformed concentration of significantly different cytokines between two cancer types at baseline, assessed by a two-sided Wilcoxon rank-sum test without adjustment for multiple comparisons. **(B)** Log_2 transformed concentration of significantly different cytokines between all four cancer types, assessed by Kruskal-Wallis test without adjustment for multiple comparisons. **(C)** Log_2 transformed concentration differences by age group for CXCL9 and IL-6 according to cancer type assessed by a two-sided Wilcoxon rank-sum test without adjustment for multiple comparisons. Box and whisker plots show the median, interquartile range (IQR), minimum/maximum values, and additional marking of outliers. Source data are provided as a Source Data file.

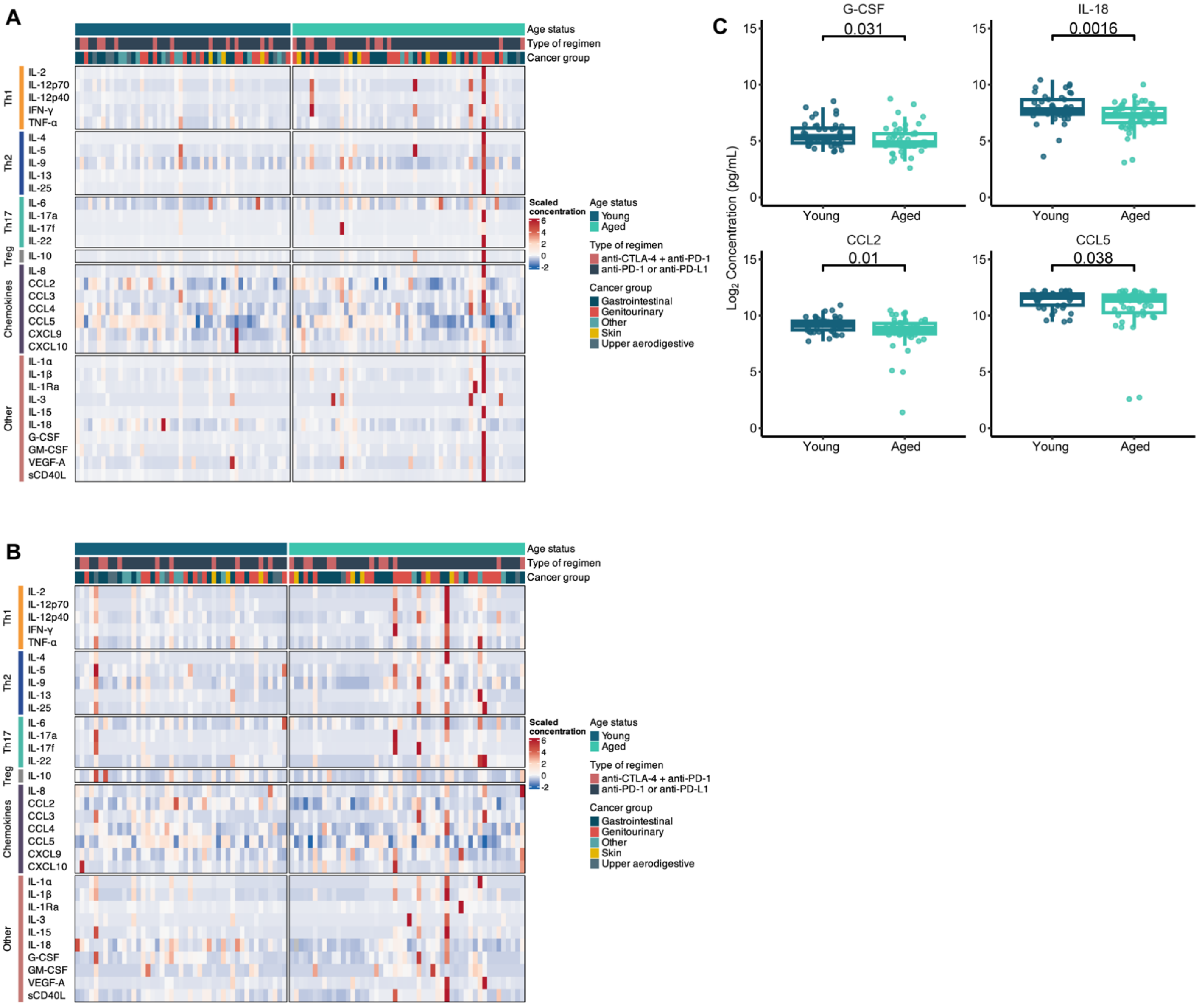

Supplementary Figure 3. Baseline and on treatment cytokine expression by patient age group. Heatmap visualization of scaled concentration of 32 cytokines grouped by age group at **(A)** baseline (n=104) and **(B)** on treatment (n=95). **(C)** Log_2 transformed concentration of significantly different cytokines by age group (aged n=50, young n=45) on treatment. Box and whisker plots show the median, interquartile range (IQR), minimum/maximum values, and additional marking of outliers. Statistical comparisons between quantitative measurements were performed using a two-sided Wilcoxon rank-sum test without adjustment for multiple comparisons. Source data are provided as a Source Data file.

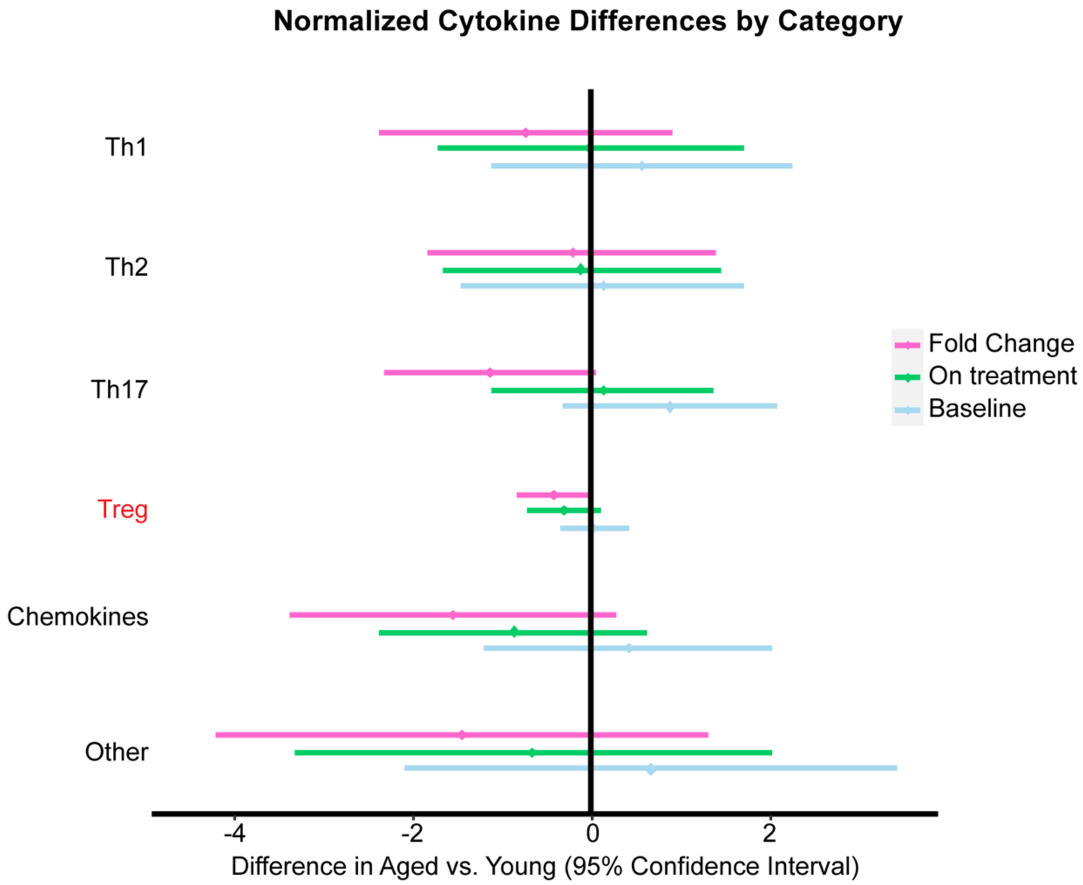

Supplementary Figure 4. Evaluation of cytokine expression as broader categories in young and aged patients.

Regression coefficient plot for age group differences in normalized cytokine levels with 95% confidence intervals. Univariate linear regression models were fitted, with age group as a predictor for each cytokine category/class. Normalized cytokine levels were calculated as the sum of z-scores within the respective cytokine category. Age was categorized into two groups, using a cutoff at 65. The coefficients indicated normalized cytokine level differences between the "Aged" group (age ≥ 65) and the "Young" group (age < 65) at time points of baseline, on-treatment, and the fold change from baseline. Normalized cytokine levels showing significant age group differences were highlighted in red. Baseline: (aged, n=54; young, n=50), On treatment/fold change (aged, n=50; young, n=45). Source data are provided as a Source Data file.

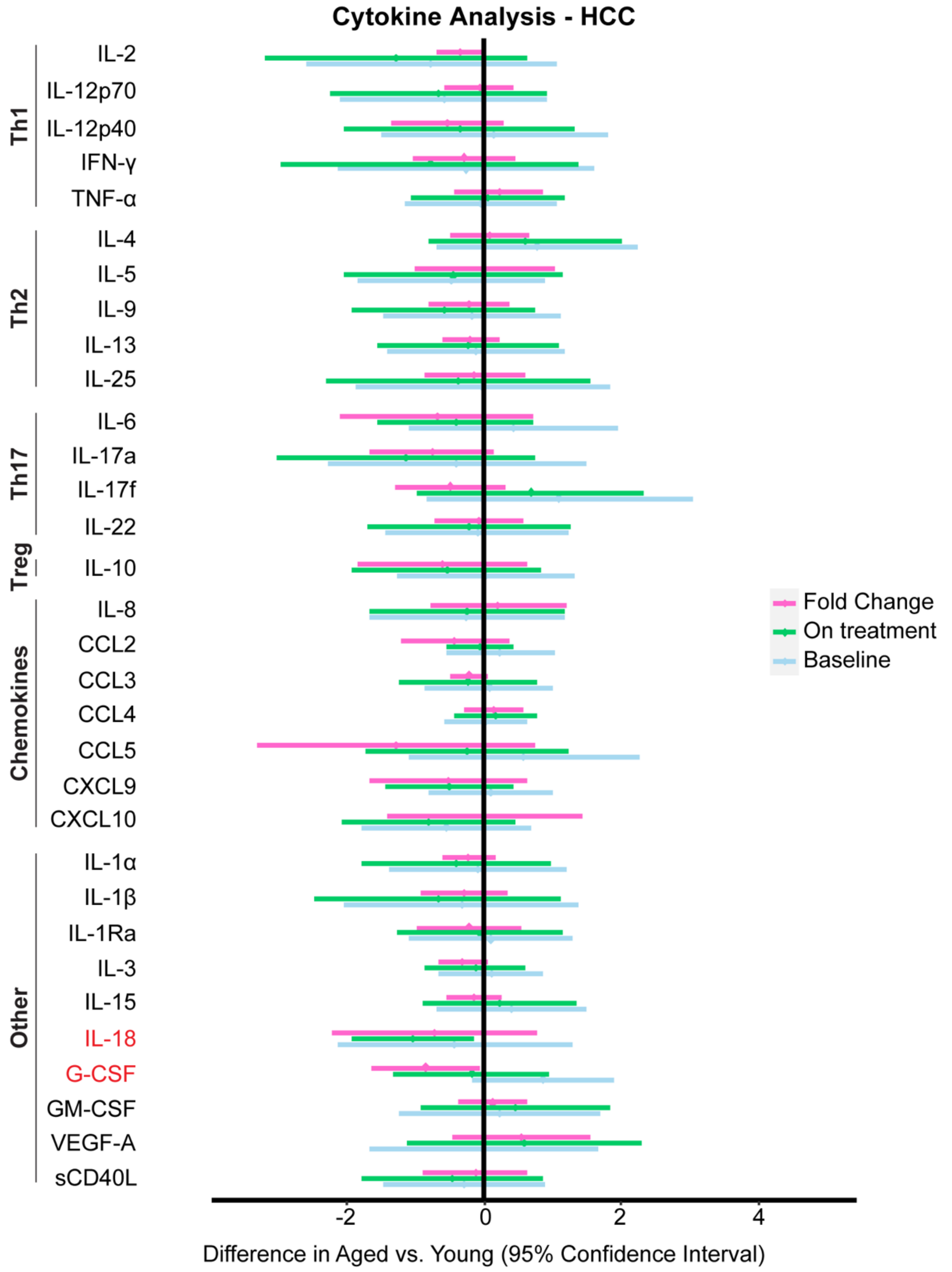

Supplementary Figure 5. Cytokine levels in patients with HCC stratified by age. Regression coefficient plot for age group differences in cytokine levels with 95% confidence intervals. Univariate linear regression models were fitted, with age group as a predictor for each cytokine outcome. Age was categorized into two groups, using a cutoff at 65. The coefficients indicated cytokine differences between the "Aged" group (age ≥ 65, n = 18) and the "Young" group (age < 65, n = 12) at time points of baseline, on-treatment, and the fold change from baseline in hepatocellular carcinoma (HCC) cohort. Cytokines showing significant age group differences were highlighted in red. Source data are provided as a Source Data file.

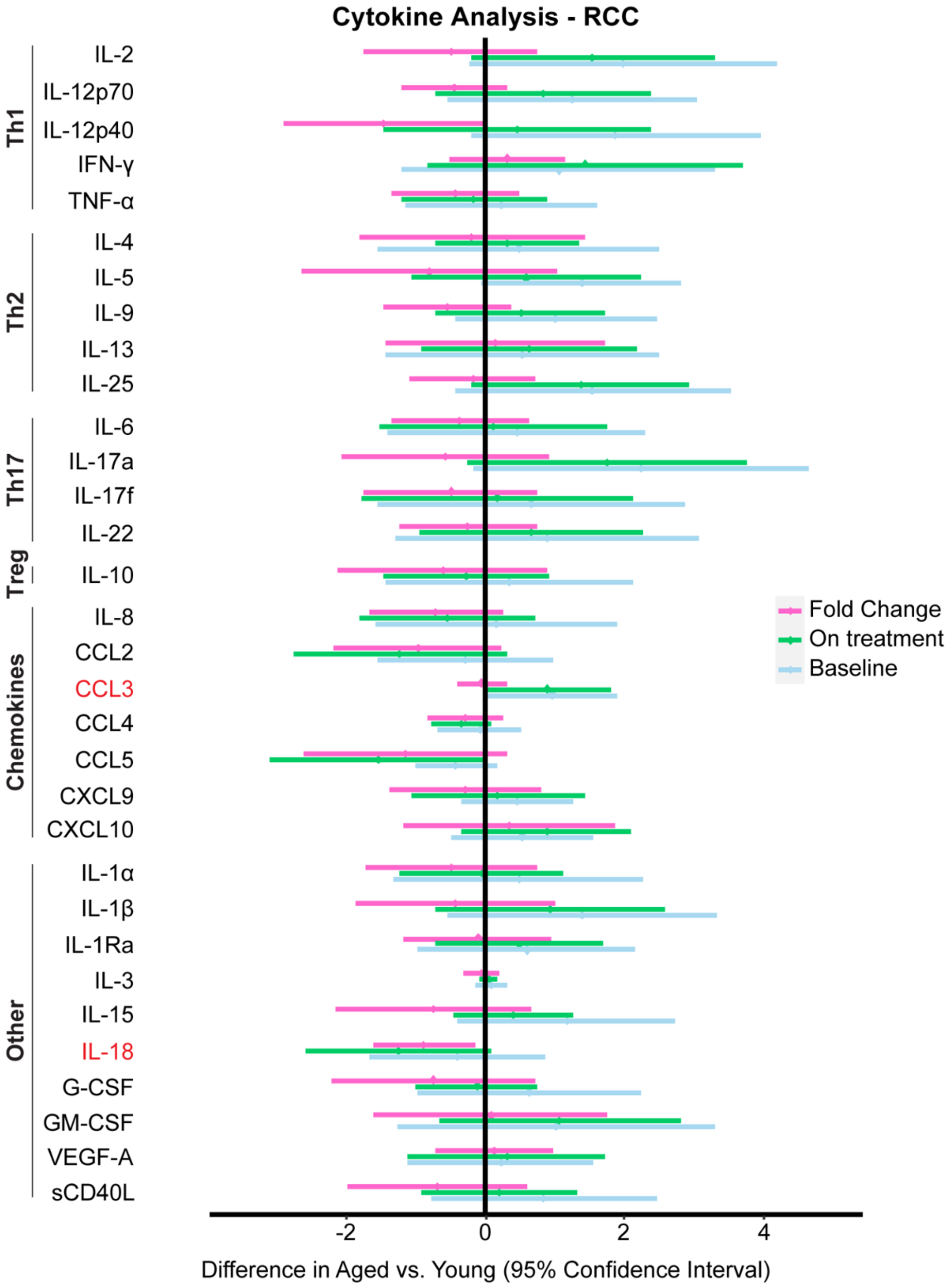

Supplementary Figure 6. Cytokine levels in patients with RCC stratified by age. Regression coefficient plot for age group differences in cytokine levels with 95% confidence intervals. Univariate linear regression models were fitted, with age group as a predictor for each cytokine outcome. Age was categorized into two groups, using a cutoff at 65. The coefficients indicated cytokine differences between the "Aged" group (age ≥ 65, n = 13) and the "Young" group (age < 65, n = 11) at time points of baseline, on-treatment, and the fold change from baseline in renal cell carcinoma (RCC) cohort. Cytokines showing significant age group differences were highlighted in red. Source data are provided as a Source Data file.

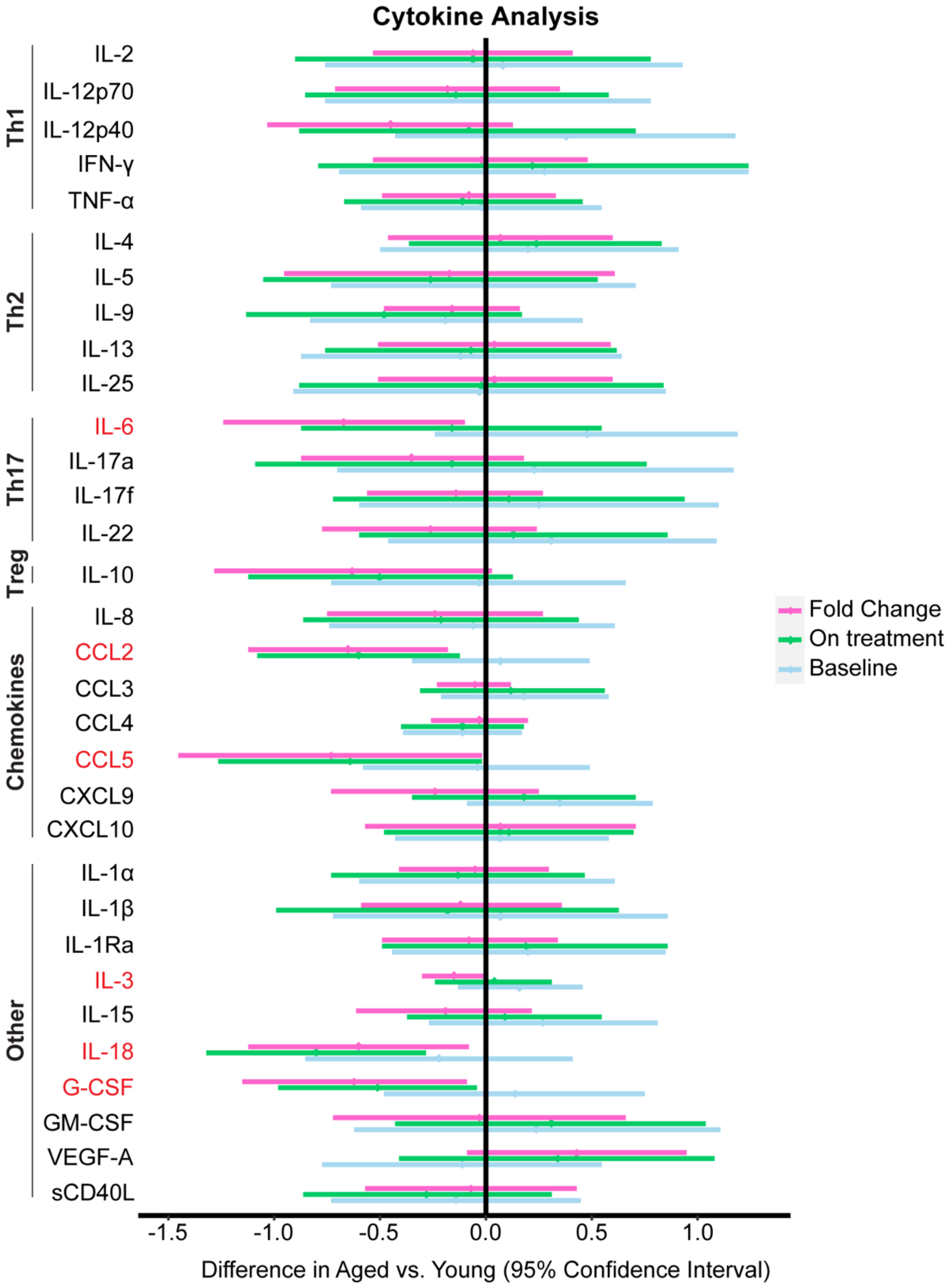

Supplementary Figure 7. Multivariable analysis of cytokine expression data across tumor types. Regression coefficient plot for age group differences in cytokine levels with 95% confidence intervals. Multivariable linear regression models were fitted, with age group adjusting for cancer group (GU vs. GI and Others vs. GI) and prior oncologic systemic therapy status (Yes vs. No) for each cytokine outcome. Age was categorized into two groups, using a cutoff at 65. The coefficients indicated cytokine level differences between the "Aged" group (age ≥ 65) and the "Young" group (age < 65) at time points of baseline, on-treatment, and the fold change from baseline. Cytokines showing significant age group differences were highlighted in red. Baseline: (aged, n=54; young, n=50), On treatment/fold change (aged, n=50; young, n=45). Source data are provided as a Source Data file.

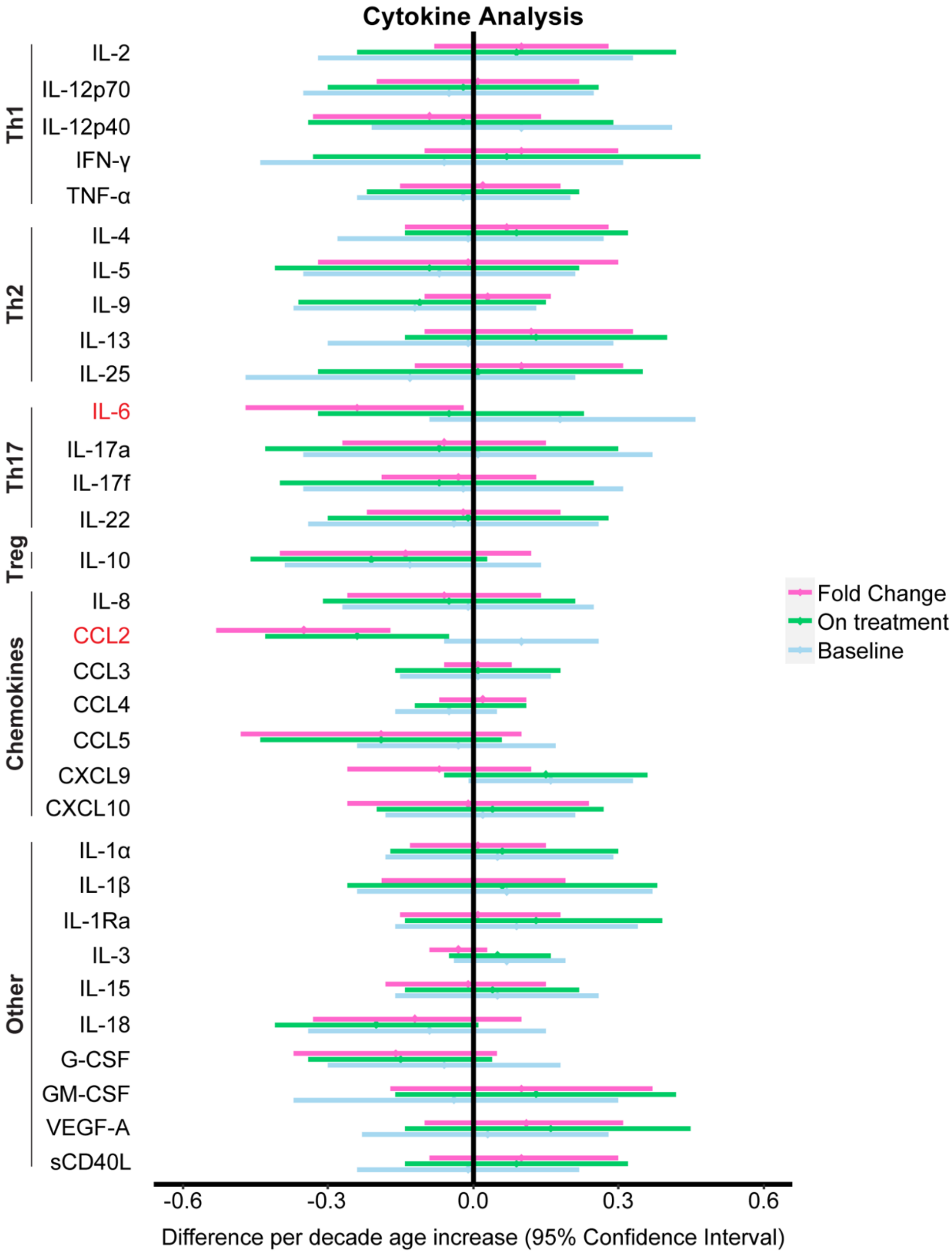

Supplementary Figure 8. Cytokine expression analysis with age as a continuous variable. Regression coefficient plot for age differences in cytokine levels with 95% confidence intervals. Multivariable linear regression models were fitted, with age adjusting for cancer group (GU vs. GI and Others vs. GI) and prior oncologic systemic therapy status (Yes vs. No) for each cytokine outcome. Age was treated as a continuous variable. The coefficients reflected cytokine level differences for each 10-year increase in age at baseline, on-treatment, and the fold change from baseline. Cytokines showing significant age differences were highlighted in red. Baseline: n=104, On treatment/fold change n=95. Source data are provided as a Source Data file.

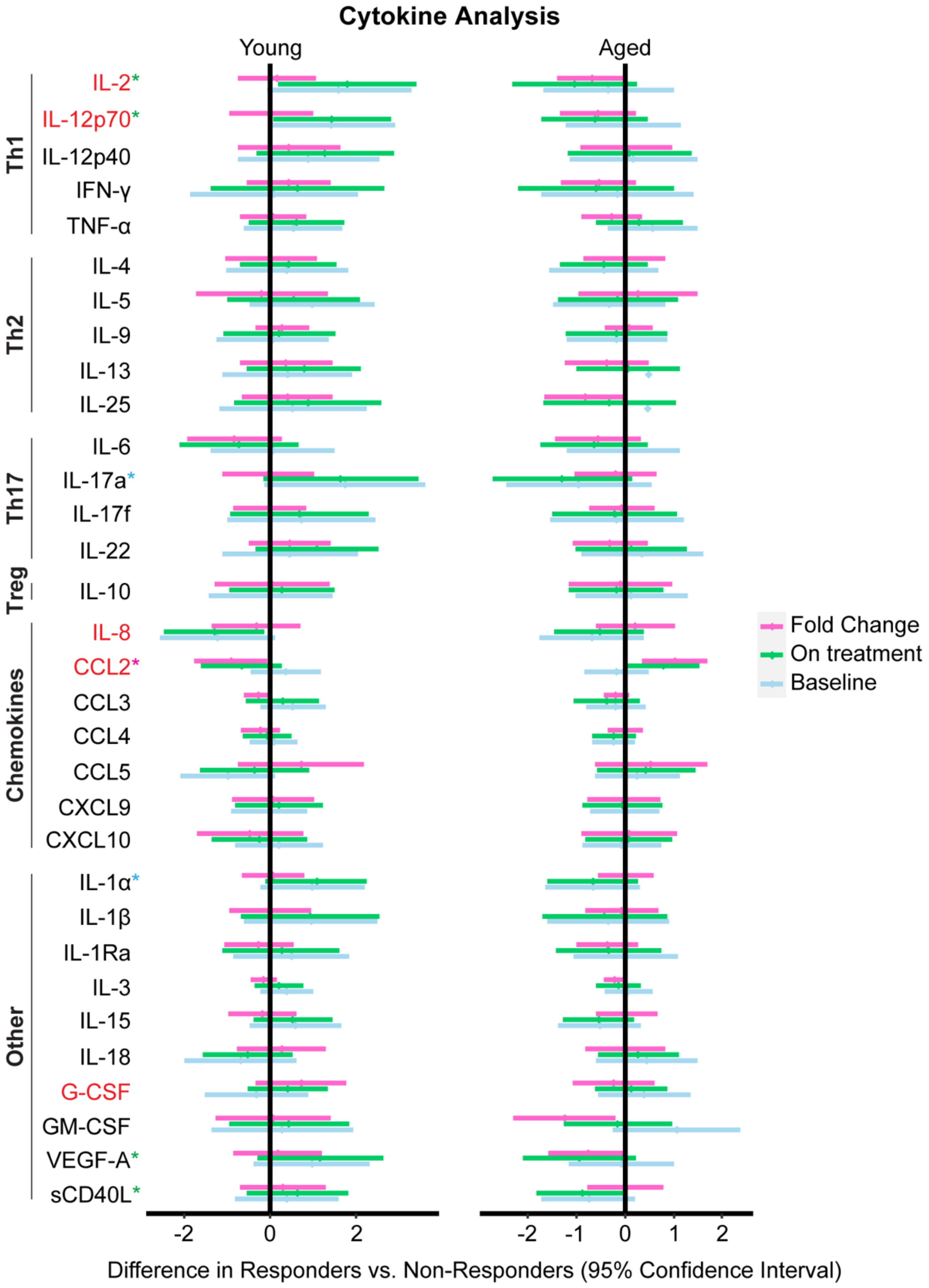

Supplementary Figure 9. Cytokine expression data and relationships with age and ICI response. Regression coefficient plot in immune response differences by age group in cytokine levels with 95% confidence intervals. Multivariable linear regression models were fitted, incorporating age group interactions with response status while adjusting for cancer group (GU vs. GI and Others vs. GI) and prior oncologic systemic therapy status (Yes vs. No) for each cytokine outcome. Age was categorized into two groups using a cutoff at 65. The coefficients reflected cytokine differences between the responders and the non-responders, for "Aged" group (age ≥ 65) and for "Young" group (age < 65), at time points of baseline (n = 88), on-treatment (n = 82), and in the fold change (n = 82) from baseline. Cytokines showing significant response group differences were highlighted in red, and those with significant interaction effects between age and immune response with multiplicity adjustment were marked with a colored (by time) asterisk. In the cytokine related to ICI response analysis, a total of 88 patients had RECIST-assessable disease and with available cytokine data at baseline. This included aged responders (n=17), aged non-responders (n=32), young responders (n=9), and young non-

responders (n=30). In the cytokine related to ICI response analysis, a total of 82 patients had RECIST-assessable disease and with available cytokine data at on-treatment time point. This included aged responders (n=17), aged non-responders (n=30), young responders (n=9), and young non-responders (n=26). Source data are provided as a Source Data file.

Characteristics	Total patients (*n* = 91)	Age ≥65 (n=49)	Age <65 (n=42)
Age on study (years)			
Median (range)	65 (20-87)	72 (65 to 87)	58 (20 to 64)
Sex - no. (%)			
Female	30 (33.0)	16 (32.7)	14 (33.3)
Male	61 (67.0)	33 (67.3)	28 (66.7)
Race - no. (%)			
White	59 (64.8)	35 (71.4)	24 (57.1)
Black	25 (27.8)	11 (22.4)	14 (33.3)
Other	7 (7.8)	3 (6.1)	4 (9.5)
Autoimmune history - no. (%)			
Yes	12 (13.2)	7 (14.3)	5 (11.9)
No	79 (86.8)	42 (85.7)	37 (88.1)
Cancer type - no. (%)			
Adrenal	1 (1.1)	0 (0.0)	1 (2.4)
Biliary Tract	2 (2.2)	1 (2.0)	1 (2.4)
Bladder	10 (11.0)	7 (14.3)	3 (7.1)
Breast	2 (2.2)	0 (0.0)	2 (4.8)
Cervical	2 (2.2)	0 (0.0)	2 (4.8)
Colorectal	1 (1.1)	1 (2.0)	0 (0.0)
Endometrial	1 (1.1)	1 (2.0)	0 (0.0)
Esophagogastric Junction	1 (1.1)	1 (2.0)	0 (0.0)
Head and Neck	7 (7.7)	2 (4.1)	5 (11.9)
Hepatocellular Carcinoma	27 (29.7)	15 (30.6)	12 (28.6)
Lung	1 (1.1)	0 (0.0)	1 (2.4)
Melanoma	3 (3.3)	1 (2.0)	2 (4.8)
Neuroendocrine	2 (2.2)	1 (2.0)	1 (2.4)
Pancreas	0 (0.0)	0 (0.0)	0 (0.0)
Renal Cell Carcinoma	23 (25.3)	13 (26.5)	10 (23.8)
Sarcoma	3 (3.3)	2 (4.1)	1 (2.4)
Squamous Cell Carcinoma of Skin	4 (4.4)	3 (6.1)	1 (2.4)
Vulvovaginal	1 (1.1)	1 (2.0)	0 (0.0)
Disease stage - no. (%)			
Early	8 (8.8)	2 (4.1)	6 (14.3)
Advanced/Metastatic	83 (91.2)	47 (95.9)	36 (85.7)
Immune checkpoint inhibitor - no. (%)			
anti-PD-1 or anti-PD-L1			
Atezolizumab	15 (16.5)	10 (20.4)	5 (11.9)
Cemiplimab	4 (4.4)	3 (6.1)	1 (2.4)
Nivolumab	13 (14.3)	6 (12.2)	7 (16.7)
Pembrolizumab	37 (40.7)	19 (38.8)	18 (42.8)
anti-CTLA-4 and anti-PD-1			
Ipilimumab + Nivolumab	22 (24.1)	11 (22.5)	11 (26.2)
Treatment regimen - no. (%)			
ICI Monotherapy	37 (40.7)	20 (40.8)	17 (40.5)
ICI Combination Therapy	22 (24.2)	11 (22.5)	11 (26.2)
ICI with Targeted Therapy or Chemotherapy	32 (35.2)	18 (36.7)	14 (33.3)
Prior systemic therapy - no. (%)			
Yes	38 (41.8)	20 (40.8)	18 (42.9)
No	53 (58.2)	29 (59.2)	24 (57.1)
Prior ICI therapy - no. (%)			
Yes	5 (5.5)	3 (6.1)	2 (4.8)
No	86 (94.5)	46 (93.9)	40 (95.2)
irAE status – no. (%)			
Grade 1 or higher irAE	48 (52.7)	30 (61.2)	18 (42.9)
No irAE	43 (47.3)	19 (38.8)	24 (57.1)
Objective Response – no. (%)	(n = 77)	(n = 45)	(n = 32)
Yes	25 (32.5)	17 (37.8)	8 (25.0)
No	52 (67.5)	28 (62.2)	24 (75.0)

Supplementary Table 2. Characteristics of the cytometry by time of flight (CyTOF) cohort. Abbreviations: ICI – immune checkpoint inhibitor; irAE – immune related adverse event, no. – number.

	≥65 years (n=49)		<65 years (n=42)		
WBC category	Mean	Std dev	Mean	Std dev	P-value
Total WBC	7.36632653	5.001999	7.8828571	4.874522	0.635709
ANC	4.99791667	4.43862	5.3316667	4.008428	0.721941
Immature granulocytes	0.05212766	0.112944	0.0605	0.122159	0.740222
ALC	1.29645833	0.614008	1.5766667	0.869291	0.191454
AMC	0.68604167	0.393845	0.6473171	0.243126	0.898428
AEC	0.1725	0.138203	0.1819512	0.144243	0.801537
CLM	1.94204082	0.837882	2.2085714	1.004227	0.253228

Supplementary Table 3. Baseline types of white blood cells based on age group.
Results of two-tailed Wilcoxon tests (*P*-value) for different types of WBC. Mean and standard deviation are reported as cells x 10^9/L. Abbreviations: AEC - absolute eosinophil count, ALC - absolute lymphocyte count, ANC - absolute neutrophil count, AMC - absolute monocyte count, CLM - combined lymphocyte and monocyte count, Std dev - standard deviation, WBC - white blood cells.

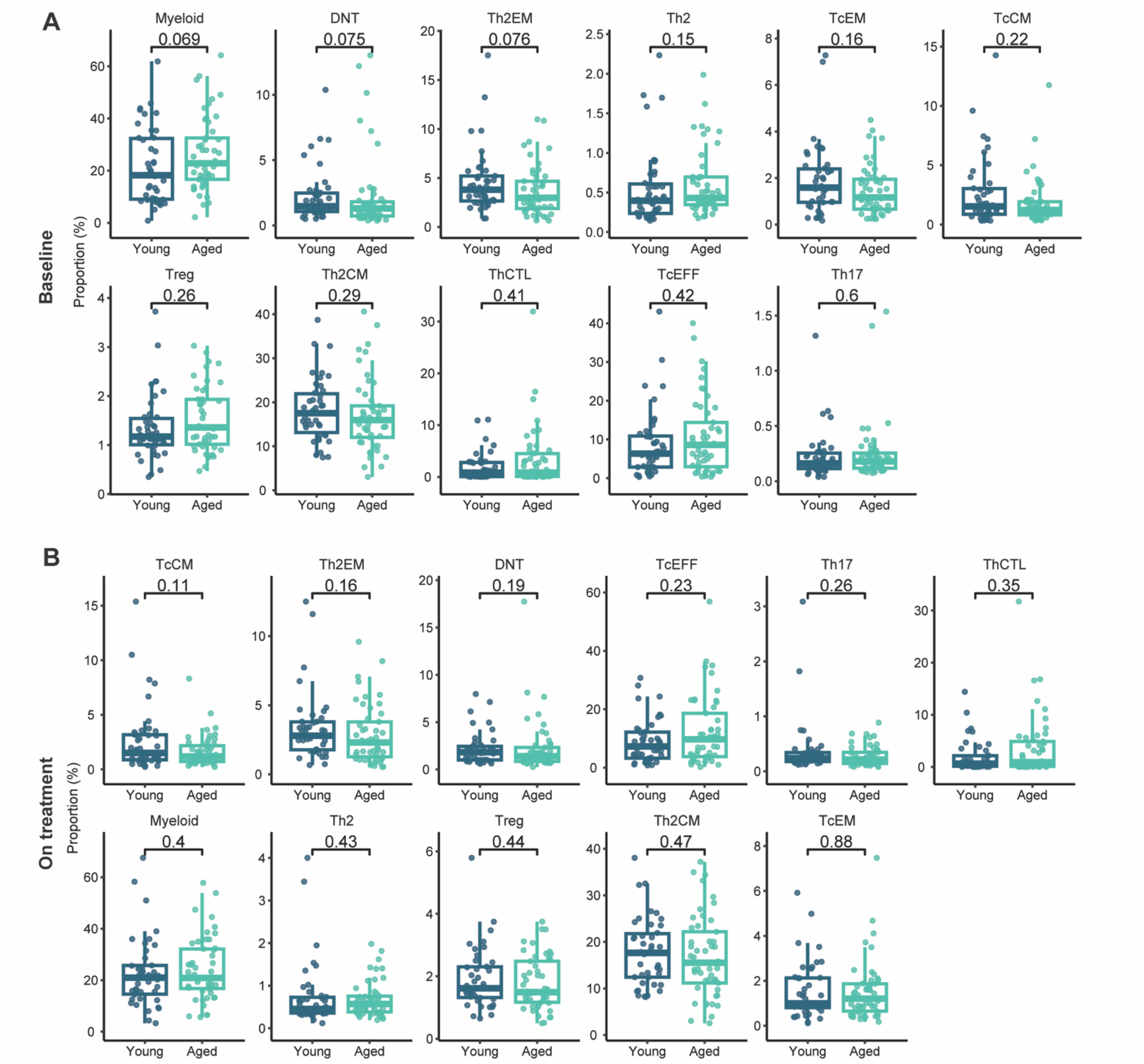

Supplementary Figure 10. Additional immune subsets in young and aged patients treated with immune checkpoint inhibitors.

Proportion of the total cells for additional major immune groups at **(A)** baseline and **(B)** on treatment (total n=91, aged n=49, young n=42). In **(A,B)**, the box and whisker plots show the median, interquartile range (IQR), minimum/maximum values, and additional marking of outliers. Statistical comparisons between quantitative measurements were performed using a two-sided Wilcoxon rank-sum test without adjustment for multiple comparisons. Source data are provided as a Source Data file.

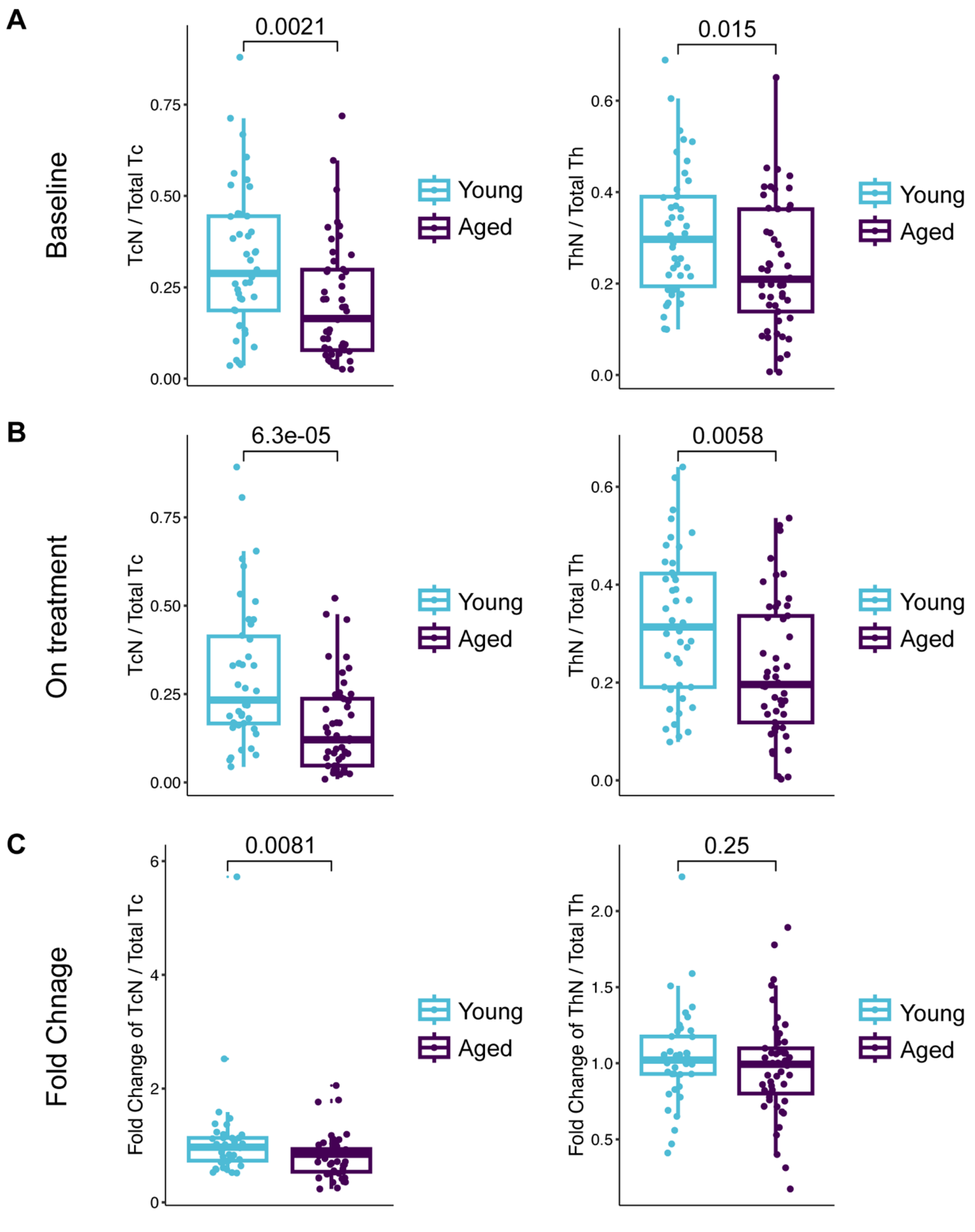

Supplementary Figure 11. Proportion of naïve cytotoxic T cells relative to total cytotoxic T cells in young and aged patients. Proportion of total TcN cells relative to parent populations for young and aged patients at (A) baseline and (B) on treatment timepoints as well as (C) fold change values (total n=91, aged n=49, young n=42). In (A-C), the box and whisker plots show the median, interquartile range (IQR), minimum/maximum values, and additional marking of outliers. Statistical comparisons between quantitative measurements were performed using a two-sided Wilcoxon rank-sum test without adjustment for multiple comparisons. Source data are provided as a Source Data file.

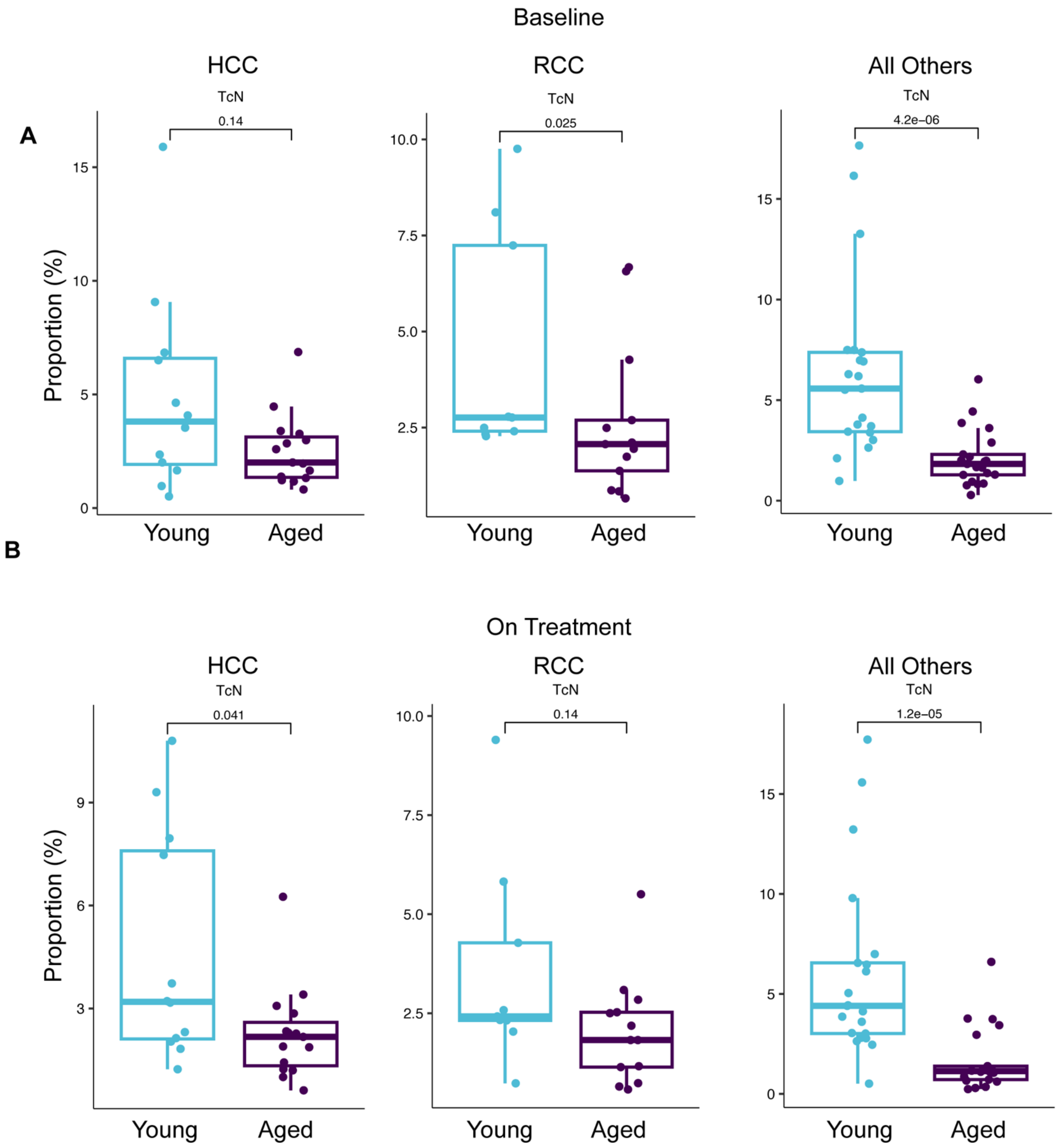

Supplementary Figure 12. **TcN cell proportion in patients with HCC, RCC, and other tumor types.** Proportion of TcN cells relative to total immune cells from cytometry by time of flight (CyTOF) analysis of patients in the cohort with either hepatocellular carcinoma (HCC), renal cell carcinoma (RCC), or any other tumor type (all others). Baseline levels (**A**) and on treatment levels (**B**) are presented. HCC: Total N=27, Young: 12, Aged: 15. RCC: Total N= 22, Young: 9, Aged: 13. All Others: Total N= 42, Young: 21, Aged = 21. The box and whisker plots show the median, interquartile range (IQR), minimum/maximum values, and additional marking of outliers. Statistical comparisons between quantitative measurements were performed using a Wilcoxon rank-sum test without adjustment for multiple comparisons. Source data are provided as a Source Data file.

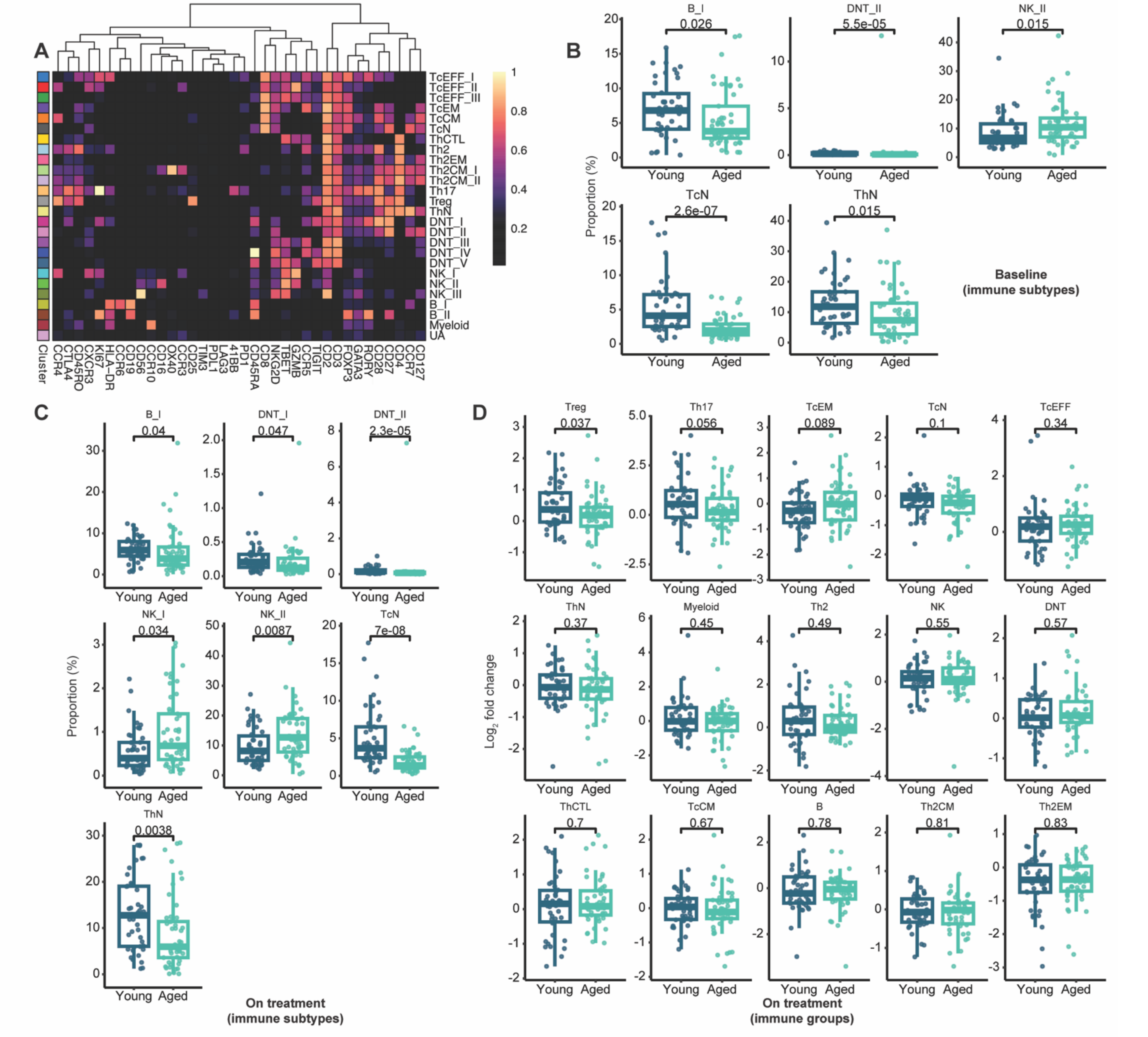

Supplementary Figure 13. Baseline and on treatment changes of 26 immune population subtypes in young and aged patients.

(A) Peripheral blood mononuclear cel (PBMC) samples were evaluated with a 37-marker cytometry by time of flight (CyTOF) panel. A FlowSOM algorithm was used to generate 35 metaclusters which were annotated into a final 26 clusters including unique subtypes of TcEFF, NK, DNT, and Th2CM cells. Proportions of selected immune subtypes by age group at (B) baseline and (C) on treatment. (D) Log_2 transformed fold change of proportion after start of ICI treatment for the 15 immune group clustering as described in Figure 3A. Box and whisker plots show the median, interquartile range (IQR), minimum/maximum values, and additional marking of outliers (total n=91, aged n=49, young n=42). Statistical comparisons between quantitative measurements were performed using a Wilcoxon rank-sum test without adjustment for multiple comparisons. Source data are provided as a Source Data file.

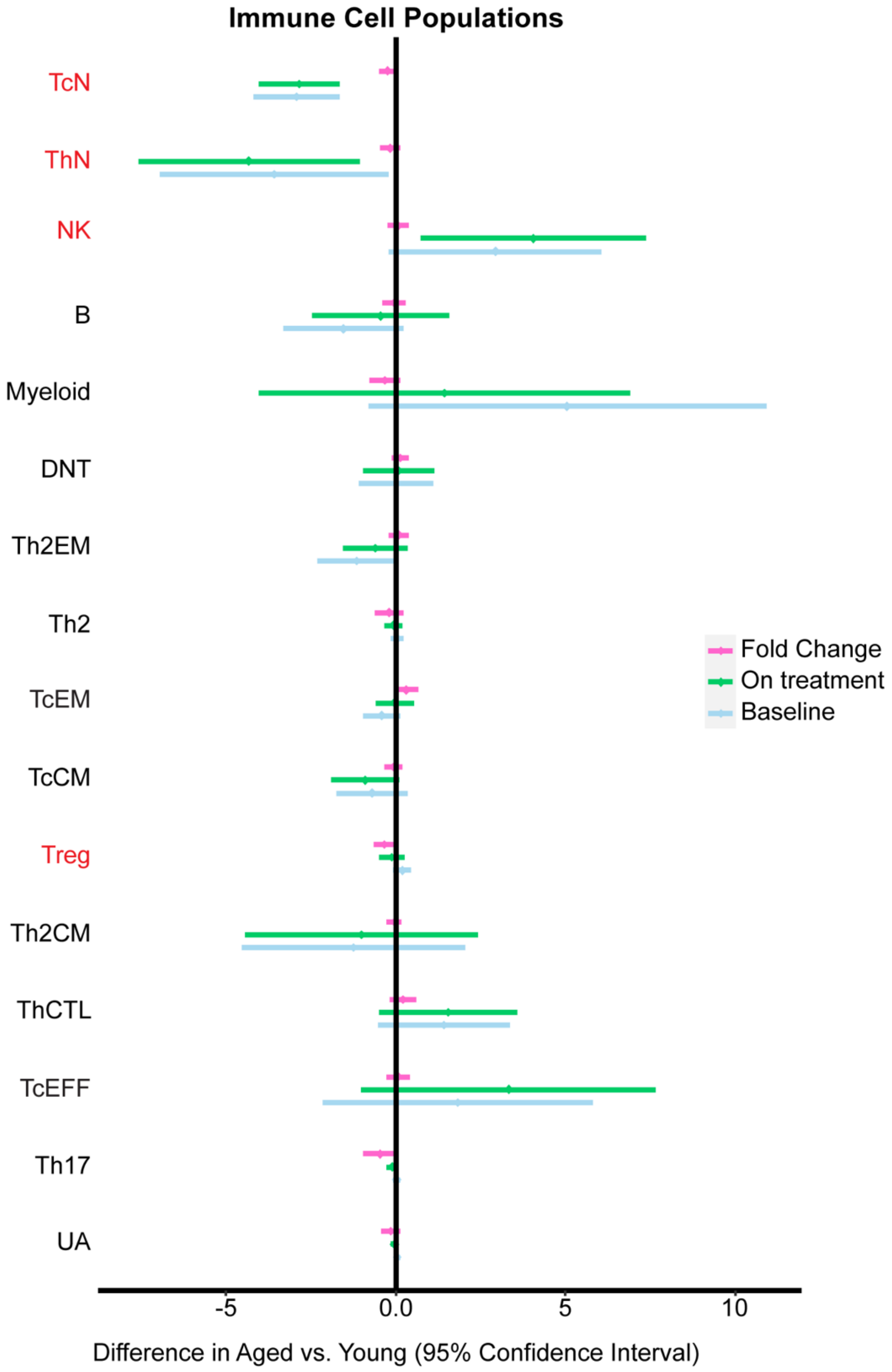

Supplementary Figure 14. Multivariable analysis of CyTOF data.
Regression coefficient plot for age group differences in clusters identified by cytometry by time of flight (CyTOF) with 95% confidence intervals. Multivariable linear regression models were fitted, with age group adjusting for cancer group (genitourinary (GU) vs. gastrointestinal (GI) and Others vs. GI) and prior oncologic systemic therapy status (Yes vs. No) for each CyTOF outcome. Age was categorized into two groups, using a cutoff at 65 (total n=91, aged n=49, young n=42). The coefficients indicated CyTOF differences between the "Aged" group (age ≥ 65) and the "Young" group (age < 65) at time points of baseline, on-treatment, and the fold change from baseline. Immune cell clusters showing significant age group differences were highlighted in red. Source data are provided as a Source Data file.

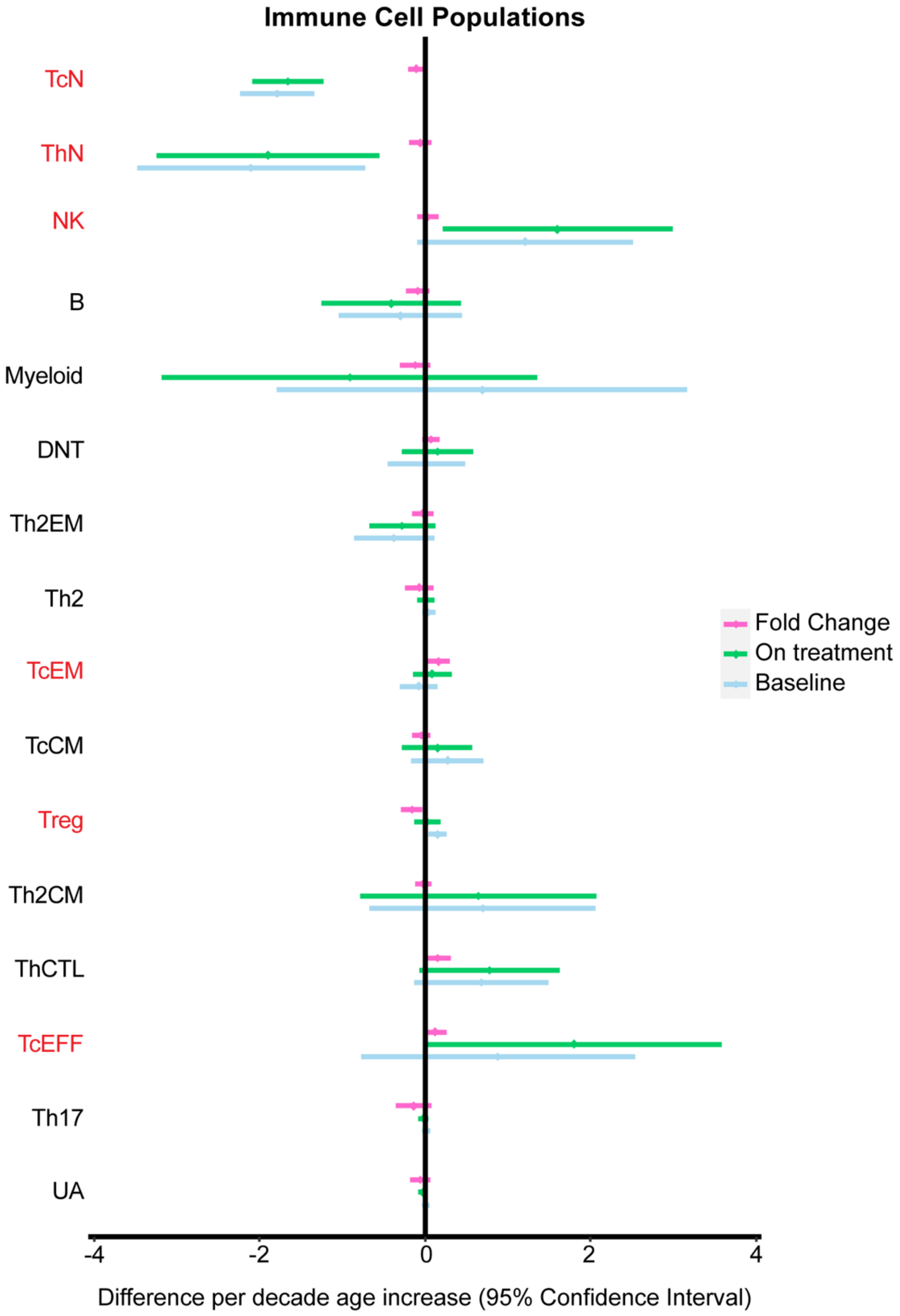

Supplementary Figure 15. CyTOF analysis with age treated as a continuous variable. Regression coefficient plot for age differences in clusters identified by cytometry by time of flight (CyTOF) with 95% confidence intervals. Multivariable linear regression models were fitted, with age adjusting for cancer group (genitourinary (GU) vs. gastrointestinal (GI) and Others vs. GI) and prior oncologic systemic therapy status (Yes vs. No) for each CyTOF outcome. Age was treated as a continuous variable (total n=91). The coefficients reflected CyTOF differences for each 10-year increase in age at baseline, on-treatment, and the fold change from baseline. Immune cell clusters showing significant age differences were highlighted in red. Source data are provided as a Source Data file.

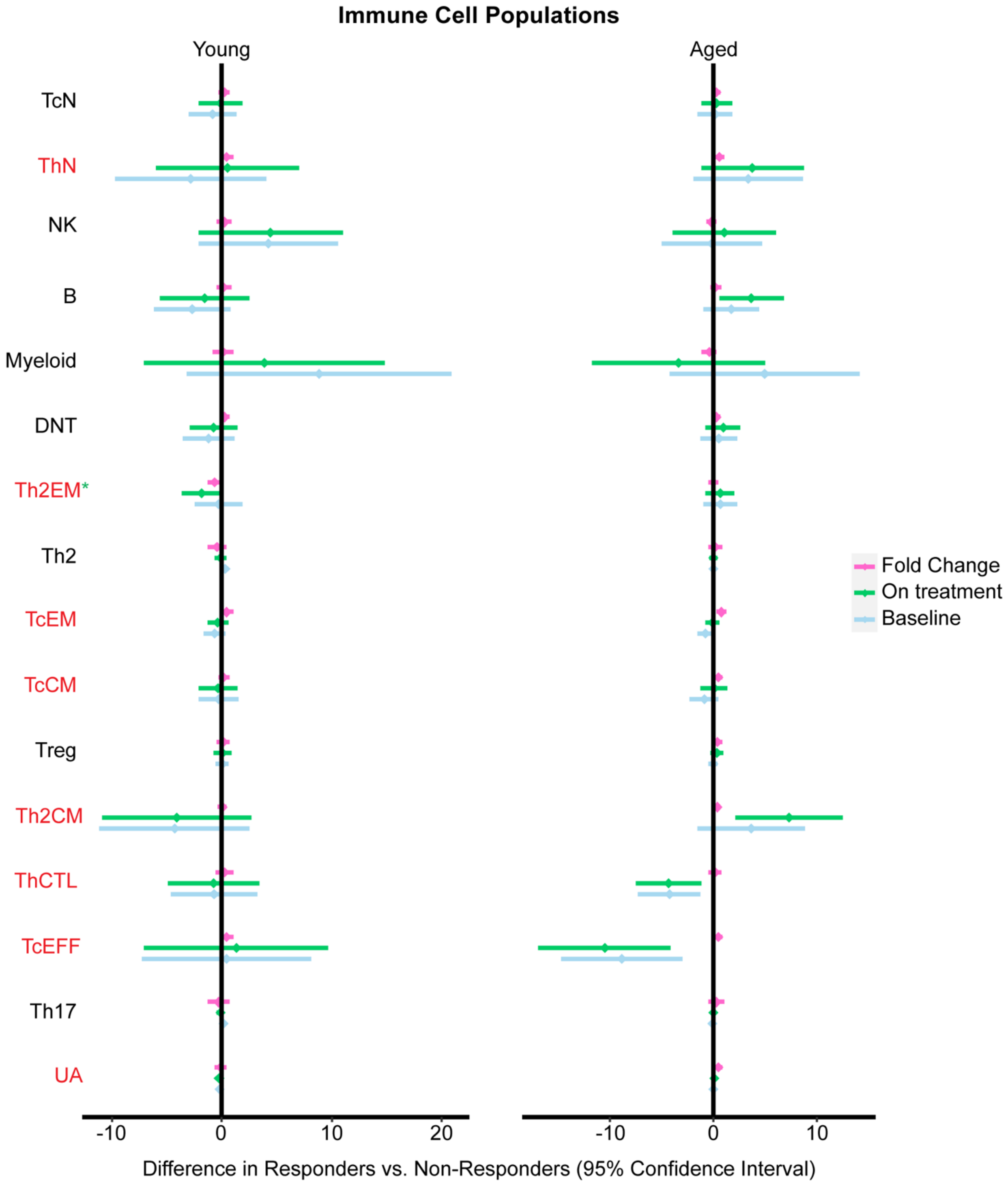

Supplementary Figure 16. Analysis of relationships between immune cell populations, age, and ICI response. Regression coefficient plot in immune response differences by age group in CyTOF cluster data with 95% confidence intervals. Multivariable linear regression models were fitted, incorporating age group interactions with response status while adjusting for cancer group (genitourinary (GU) vs. gastrointestinal (GI) and Others vs. GI) and prior oncologic systemic therapy status (Yes vs. No) for each cytometry by time of flight (CyTOF) cluster. Age was categorized into two groups using a cutoff at 65. The coefficients reflected CyTOF differences between the responders and the non-responders, for "Aged" group (age ≥ 65) and for "Young" group (age < 65) stratified by immune checkpoint inhibitor (ICI) response (aged responders (n=17), aged non-responders (n=29), young responders (n=8), and young non-responders (n=24), at time points of baseline, on-treatment, and in the fold change from baseline. Immune cell clusters showing significant response group

differences were highlighted in red. All interaction effects between age and immune response were non-significant after multiplicity adjustment. Source data are provided as a Source Data file.

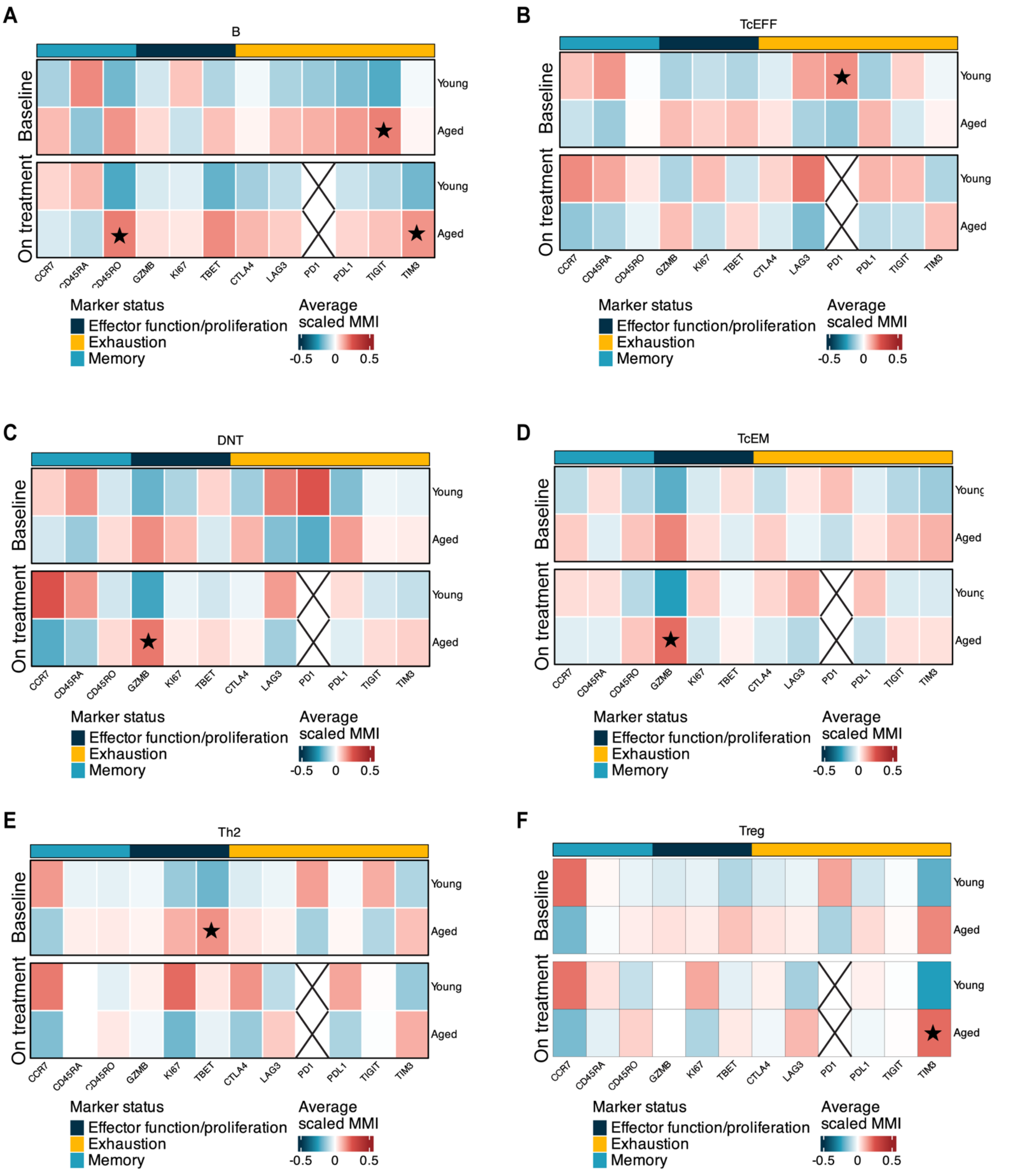

Supplementary Figure 17. Functional marker expression in non-naïve cell subsets by age.
Heatmaps were generated for the average scaled mean metal intensity (MMI) between aged (n=49) and young patients (n=42) for 12 markers of interest related to T cell memory, effector/proliferation function, and exhaustion

for 15 unique immune clusters at baseline and on treatment. Scaled MMIs were calculated for each individual marker within each immune cluster as defined from the annotation clustering from Figure 3A. Scaling was performed for visualization purposes to highlight the most divergent markers by age group, and formal statistical comparisons were performed with a two-sided Wilcoxon rank-sum test on non-scaled MMIs without adjustment for multiple comparisons, with statistically significant comparisons ($P < 0.05$) indicated on the heatmaps with a star in the box of the more highly expressed marker. Exact P values are reported in a Source Data file. Analysis of PD-1 (PD1) expression in post-treatment samples was not included due to use of a competitive antibody as described in the methods. (A-F) represents selected non-naïve immune clusters. Source data are provided as a Source Data file.

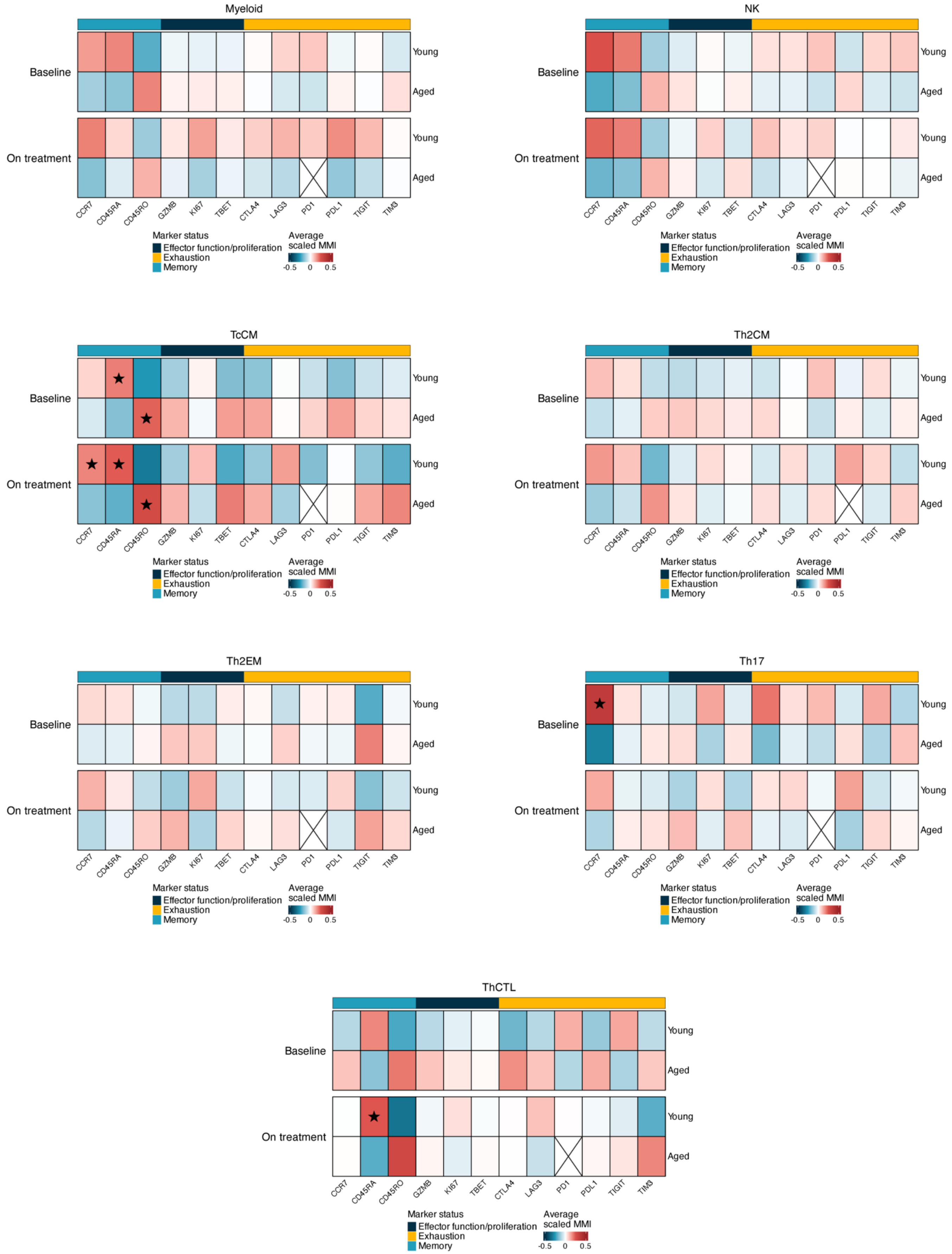

Supplementary Figure 18. Baseline and on treatment marker differences within additional immune cell clusters.

Heatmaps were generated for the average scaled mean metal intensity (MMI) between aged (n=49) and young patients (n=42) for 12 markers of interest related to T cell memory, effector/proliferation function, and exhaustion for 15 unique immune clusters at baseline and on treatment for additional immune cell clusters not included in Figure 5. Scaling was performed for visualization purposes to highlight the most divergent markers by age group, and formal statistical comparisons were performed with a two-sided Wilcoxon rank-sum test on non-scaled MMIs without adjustment for multiple comparisons, with statistically significant comparisons ($P < 0.05$) indicated on the heatmaps with a star in the box of the more highly expressed marker. Exact P values are reported in a Source Data file. Analysis of PD-1 (PD1) expression in post-treatment samples was not included due to use of a competitive antibody as described in the methods. Source data are provided as a Source Data file.

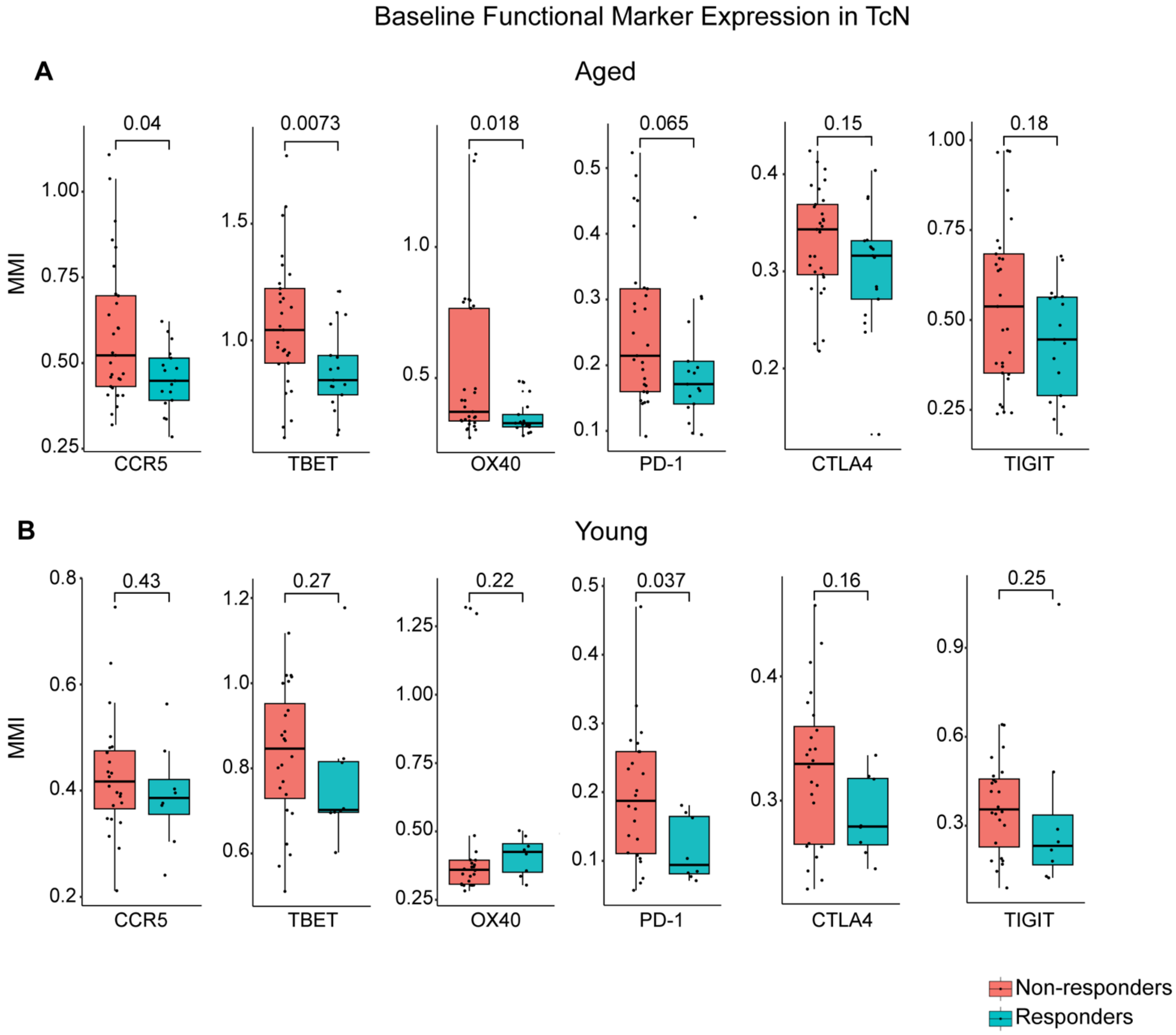

Supplementary Figure 19. Functional marker expression in TcN cells by age.
Baseline functional marker expression assessed by mean metal intensity (MMI) in TcN cells from (A) aged responders (n=17) and aged non-responders (n=28) and (B) young responders (n=8) and young non-responders (n=24). Box and whisker plots showing the median, interquartile range (IQR), and minimum/maximum values. Statistical comparisons were performed with a two-sided Wilcoxon rank-sum test without adjustment for multiple comparisons. Source data are provided as a Source Data file.
