# Supplementary material for: Age-related divergence of circulating immune responses in patients with solid tumors treated with immune checkpoint inhibitors
Source: Nat Commun. 2025 Apr 21;16:3531. doi: 10.1038/s41467-025-58512-z (PMC12012091; doi:10.1038/s41467-025-58512-z)
Supplement: Supplementary file 2 — Description of Additional Supplementary Files [file 41467_2025_58512_MOESM2_ESM.pdf]

Description of Additional Supplementary Files

Supplementary Data 1 – List and description of antibodies included in the Cytometry by Time-of-Flight (CyTOF) panel.

Supplementary Data 2 – Definition of clusters based on marker expression from Cytometry by Time-of-Flight (CyTOF) staining and analysis.
