# Supplementary material for: Age-related divergence of circulating immune responses in patients with solid tumors treated with immune checkpoint inhibitors
Source: Nat Commun. 2025 Apr 21;16:3531. doi: 10.1038/s41467-025-58512-z (PMC12012091; doi:10.1038/s41467-025-58512-z)
Supplement: Supplementary file 5 — Reporting Summary [file 41467_2025_58512_MOESM5_ESM.pdf]

nature portfolio

Corresponding author(s): Daniel Zabransky
Mark Yarchoan
Won Jin Ho

Last updated by author(s): Feb 5, 2025

Reporting Summary

Nature Portfolio wishes to improve the reproducibility of the work that we publish. This form provides structure for consistency and transparency in reporting. For further information on Nature Portfolio policies, see our Editorial Policies and the Editorial Policy Checklist.

Statistics

For all statistical analyses, confirm that the following items are present in the figure legend, table legend, main text, or Methods section.

n/a	Confirmed	
☐	☒	The exact sample size (*n*) for each experimental group/condition, given as a discrete number and unit of measurement
☐	☒	A statement on whether measurements were taken from distinct samples or whether the same sample was measured repeatedly
☐	☒	The statistical test(s) used AND whether they are one- or two-sided<br>*Only common tests should be described solely by name; describe more complex techniques in the Methods section.*
☐	☒	A description of all covariates tested
☐	☒	A description of any assumptions or corrections, such as tests of normality and adjustment for multiple comparisons
☐	☒	A full description of the statistical parameters including central tendency (e.g. means) or other basic estimates (e.g. regression coefficient) AND variation (e.g. standard deviation) or associated estimates of uncertainty (e.g. confidence intervals)
☐	☒	For null hypothesis testing, the test statistic (e.g. *F*, *t*, *r*) with confidence intervals, effect sizes, degrees of freedom and *P* value noted<br>*Give P values as exact values whenever suitable.*
☒	☐	For Bayesian analysis, information on the choice of priors and Markov chain Monte Carlo settings
☒	☐	For hierarchical and complex designs, identification of the appropriate level for tests and full reporting of outcomes
☒	☐	Estimates of effect sizes (e.g. Cohen's *d*, Pearson's *r*), indicating how they were calculated

Our web collection on statistics for biologists contains articles on many of the points above.

Software and code

Policy information about availability of computer code

Data collection

Key Resources

Software and R packages Source Identifier
Cairo R package Urbanek S, Horner J (2022). _Cairo: R Graphics Device using Cairo Graphics Library for Creating High-Quality Bitmap (PNG, JPEG, TIFF), Vector (PDF, SVG, PostScript) and Display (X11 and Win32) Output_. https://CRAN.R-project.org/package=Cairo
circlize R package Gu, Z. (2014) circlize implements and enhances circular visualization in R. Bioinformatics. https://cran.r-project.org/web/packages/circlize/index.html
ComplexHeatmap R package Gu, Z. (2016) Complex heatmaps reveal patterns and correlations in multidimensional genomic data. Bioinformatics. https://CRAN.R-project.org/package=ComplexHeatmap
ConsensusClusterPlus R package Wilkerson, D. M, Hayes, Neil D (2010). "ConsensusClusterPlus: a class discovery tool with confidence assessments and item tracking." Bioinformatics, 26(12), 1572-1573. https://bioconductor.org/packages/release/bioc/html/ConsensusClusterPlus.html

CytoNorm Van Gassen S, Gaudilliere B, Angst MS, Saeys Y, Aghaeepour N. CytoNorm: A Normalization Algorithm for Cytometry Data. Cytom Part J Int Soc Anal Cytol. 2020;97(3):268-278. doi:10.1002/cyto.a.23904 https://github.com/saeyslab/CytoNorm

dplyr R package Wickham H, François R, Henry L, Müller K, Vaughan D (2023). _dplyr: A Grammar of Data Manipulation_. https://CRAN.R-project.org/package=dplyr
flowCore R package Ellis B, Haaland P, Hahne F, Le Meur N, Gopalakrishnan N, Spidlen J, Jiang M, Finak G (2023). flowCore: flowCore: Basic structures for flow cytometry data. https://bioconductor.org/packages/release/bioc/html/flowCore.html

FlowSOM R package Van Gassen S, Callebaut B, Van Helden M, Lambrecht B, Demeester P, Dhaene T, Saeys Y (2015). "FlowSOM: Using self-organizing maps for visualization and interpretation of cytometry data." Cytometry Part A, 87(7), 636-645. https://bioconductor.org/packages/release/bioc/html/FlowSOM.html

ggplot2 R package H. Wickham. ggplot2: Elegant Graphics for Data Analysis. Springer-Verlag New York, 2016. https://ggplot2.tidyverse.org
ggpubr R package Kassambara A (2023). _ggpubr: 'ggplot2' Based Publication Ready Plots_. https://cran.r-project.org/web/packages/ggpubr/index.html

ggrepel R package Slowikowski K (2023). _ggrepel: Automatically Position Non-Overlapping Text Labels with 'ggplot2'_. https://cran.r-project.org/web/packages/ggrepel/index.html

ggridges R package Wilke C (2022). _ggridges: Ridgeline Plots in 'ggplot2'_. https://cran.r-project.org/web/packages/ggridges/index.html

ggsci R package Xiao N (2023). _ggsci: Scientific Journal and Sci-Fi Themed Color Palettes for 'ggplot2'_. https://CRAN.R-project.org/package=ggsci
here R package Müller K (2020). _here: A Simpler Way to Find Your Files_. https://CRAN.R-project.org/package=here
Hmisc R package Harrell Jr F (2023). _Hmisc: Harrell Miscellaneous_. https://cran.r-project.org/web/packages/Hmisc/index.html

khroma R package Frerebeau N (2024). _khroma: Colour Schemes for Scientific Data Visualization_. Université Bordeaux
Montaigne, Pessac, France. doi:10.5281/zenodo.1472077
https://packages.tesselle.org/khroma/
limma R package Ritchie ME, Phipson B, Wu D, Hu Y, Law CW, Shi W, Smyth GK (2015). "limma powers differential expression analyses for RNA-sequencing and microarray studies." Nucleic Acids Research, 43(7), e47. https://bioconductor.org/packages/release/bioc/html/limma.html

matrixStats R package Bengtsson H (2023). _matrixStats: Functions that Apply to Rows and Columns of Matrices (and to Vectors)_. https://cran.rstudio.com/web/packages/matrixStats/index.html

NatParksPalettes Blake K (2022). _NatParksPalettes: Color Palettes Inspired by National Parks_. R package version 0.2.0 https://CRAN.R-project.org/package=NatParksPalettes
openxlsx R package Schauberger P, Walker A (2023). _openxlsx: Read, Write and Edit xlsx Files_. https://CRAN.R-project.org/package=openxlsx
pals R package Wright K (2023). _pals: Color Palettes, Colormaps, and Tools to Evaluate Them_. https://cran.r-project.org/web/packages/pals/index.html

pheatmap R package Kolde R (2019). _pheatmap: Pretty Heatmaps_. https://cran.r-project.org/web/packages/pheatmap/index.html

Prism v10 GraphPad https://www.graphpad.com/
randomcoloR R package Ammar R (2019). _randomcoloR: Generate Attractive Random Colors_. https://cran.r-project.org/web/packages/randomcoloR/index.html

raster R package Hijmans R (2023). _raster: Geographic Data Analysis and Modeling_. https://cran.r-project.org/web/packages/raster/index.html

RColorBrewer R package Neuwirth E (2022). _RColorBrewer: ColorBrewer Palettes_. https://cran.r-project.org/web/packages/RColorBrewer/index.html

readxl R package Wickham H, Bryan J (2023). _readxl: Read Excel Files_. https://cran.r-project.org/web/packages/readxl/index.html

reshape2 R package Hadley Wickham (2007). Reshaping Data with the reshape Package. Journal of Statistical Software, 21(12), 1-20. https://cran.r-project.org/web/packages/reshape2/index.html

scales R package Wickham H, Seidel D (2022). _scales: Scale Functions for Visualization_. https://cran.r-project.org/web/packages/scales/index.html

stringr R package Wickham H (2022). _stringr: Simple, Consistent Wrappers for Common String Operations_. https://CRAN.R-project.org/package=stringr
survival R package Therneau T (2023). _A Package for Survival Analysis in R_. https://CRAN.R-project.org/package=survival
survminer R package Kassambara A, Kosinski M, Biecek P (2021). _survminer: Drawing Survival Curves using 'ggplot2'_. https://CRAN.R-project.org/package=survminer
tidyverse R package Wickham H, et. al (2019). "Welcome to the tidyverse." _Journal of Open Source Software_, *4*(43), 1686. doi:10.21105/joss.01686 https://doi.org/10.21105/joss.01686
umap R package Konopka T (2023). _umap: Uniform Manifold Approximation and Projection_. https://cran.r-project.org/web/packages/umap/index.html

viridis R package Simon Garnier, Noam Ross, Robert Rudis, Antônio P. Camargo, Marco Sciaini, and Cédric Scherer (2023). viridis(Lite) - Colorblind-Friendly Color Maps for R. viridis package version 0.6.4. https://CRAN.R-project.org/package=viridis

Data analysis

Code will be deposited to GitHub at https://github.com/dzabran1/ICI-Aging.git

For manuscripts utilizing custom algorithms or software that are central to the research but not yet described in published literature, software must be made available to editors and reviewers. We strongly encourage code deposition in a community repository (e.g. GitHub). See the Nature Portfolio guidelines for submitting code & software for further information.

Data

Policy information about availability of data

All manuscripts must include a data availability statement. This statement should provide the following information, where applicable:

- Accession codes, unique identifiers, or web links for publicly available datasets
- A description of any restrictions on data availability
- For clinical datasets or third party data, please ensure that the statement adheres to our policy

De-identified CyTOF data files will be deposited and available at Zenodo (https://doi.org/10.5281/zenodo.14755936). The authors declare that the minimal data set for this study cannot be shared publicly due to ethical and legal restrictions on sharing de-identified data that aligns with the consent of research participants. Current JHU compliance policies require data with no direct consent for public open access sharing be under restricted access. Access may be obtained through Vivli, an established repository for clinical data that provides open access without a fee restricted to approved researchers under a Data Use Agreement. JHU compliance policy for Vivli requires additional anonymization of certain demographics, including use of age ranges and limiters to outlier values for weight, height, and certain rare diseases, while retaining sufficient value for reference and validation of results. Researchers can request more detailed data from the corresponding author shared though an approved collaboration arrangement.

Research involving human participants, their data, or biological material

Policy information about studies with human participants or human data. See also policy information about sex, gender (identity/presentation), and sexual orientation and race, ethnicity and racism.

Reporting on sex and gender	Sex of the patients was determined based on self-reporting by patients in the electronic medical record for those patients enrolled in the clinical trial. Discrete sex-based analyses were not performed as the size of the resulting sex-stratified cohorts were relatively small. Overall composition of the age-stratified cohorts did not differ based on sex of the patients (Table 1).
Reporting on race, ethnicity, or other socially relevant groupings	Self-reported race categories were extracted from the electronic medical record for the patients included in this study. In this study, patients are reported as identifying as white, black, or other (if they chose another option that was not white or black). Participants selected from the categories listed below:<br>• white<br>• black<br>• hawaiian or pacific islander<br>• asian<br>• native american or alsakan<br>• two or more races, not reported<br>• other<br>• unknown<br>• declined to answer
Population characteristics	The patient cohort was stratified by patient age (65 years of age or older at diagnosis or under 65 years of age at diagnosis). Table 1 lists the clinical characteristics of the patients included in this study.
Recruitment	We are conducting an ongoing prospective observational study of patients with solid tumors who received ICI treatment as standard of care at Johns Hopkins University from May 2021 to the present. Patients included in this data analysis were enrolled from May 2021 to October 2022, and data censorship was set at 6 months from last consented patient included in the analysis (April 2023). Eligible patients were aged > 18 years with pathologically confirmed solid tumors treated with ICIs consisting of anti-PD-1/PD-L1 blockade (nivolumab, pembrolizumab, atezolizumab, cemiplimab, durvalumab, and avelumab), combination ICI blockade with anti-PD-1 (nivolumab) and anti-CTLA-4 (ipilimumab), or in combination with targeted therapy or chemotherapy. All patients enrolled in the study had peripheral blood samples collected at baseline prior to initiation of the ICI. Subsequently, peripheral blood samples were collected at month 1, 2, 4, 6, and 12 as long as the patient was continued on ICI and if available. Information regarding tumor molecular biomarkers of ICI response including tumor mutational burden (TMB), PD-L1, and high microsatellite instability (MSI-H) and mismatch repair deficient (dMMR) status, was available for a subset of patients who had undergone testing with a commercially available molecular profiling assay (including but not limited to Tempus, Caris, and Foundation Medicine) as standard of care. PD-L1 classification (none, low, high) was based on the assay utilized. If a patient had multiple types of PD-L1 assessments such as tumor proportion score (TPS) and combined positive score (CPS), then the PD-L1 value used for classification was the type of PD-L1 assessment utilized for that specific tumor type.
Ethics oversight	The study protocol was approved by the Johns Hopkins Institutional Review Board (IRB #00267960), and all participants provided written informed consent before the blood samples and clinical data were collected.

Note that full information on the approval of the study protocol must also be provided in the manuscript.

Field-specific reporting

Please select the one below that is the best fit for your research. If you are not sure, read the appropriate sections before making your selection.

☒ Life sciences ☐ Behavioural & social sciences ☐ Ecological, evolutionary & environmental sciences

For a reference copy of the document with all sections, see nature.com/documents/nr-reporting-summary-flat.pdf

Life sciences study design

All studies must disclose on these points even when the disclosure is negative.

Sample size	Sample size was not predetermined, but was set based on enrollment in the clinical trial during the specified study period. With a sample size of 104 patients included in this analysis, there is 82% power to detect an approximately 0.5 standard deviation difference between groups with an alpha of 0.05.
Data exclusions	For the analysis in Supplementary Figure 19, one patient from the CyTOF cohort who had response information was not included as they did not have cells assigned to the defined CyTOF cluster corresponding to those in the T cell phenotyping analysis.
Replication	Cytokine assays on samples were conducted across multiple batches. To account for batch effect and ensure accurate fold change calculations, on-treatment samples of each patient were always run with the corresponding baseline sample. For patients with baseline samples that were run in multiple batches, the average concentration of those baseline samples was used in baseline analyses.
Randomization	This study did not involve randomization. Participants were allocated into the experimental groups based on age at diagnosis. The median age of the cohort was approximately 65 years old and patients were stratified as 65 or older or under 65 years of age.
Blinding	Investigators performing cytokine measurement assays and CyTOF staining protocols were blinded to participant age group status during data collection and initial analysis.

Behavioural & social sciences study design

All studies must disclose on these points even when the disclosure is negative.

Study description	*Briefly describe the study type including whether data are quantitative, qualitative, or mixed-methods (e.g. qualitative cross-sectional, quantitative experimental, mixed-methods case study).*
Research sample	*State the research sample (e.g. Harvard university undergraduates, villagers in rural India) and provide relevant demographic information (e.g. age, sex) and indicate whether the sample is representative. Provide a rationale for the study sample chosen. For studies involving existing datasets, please describe the dataset and source.*
Sampling strategy	*Describe the sampling procedure (e.g. random, snowball, stratified, convenience). Describe the statistical methods that were used to predetermine sample size OR if no sample-size calculation was performed, describe how sample sizes were chosen and provide a rationale for why these sample sizes are sufficient. For qualitative data, please indicate whether data saturation was considered, and what criteria were used to decide that no further sampling was needed.*
Data collection	*Provide details about the data collection procedure, including the instruments or devices used to record the data (e.g. pen and paper, computer, eye tracker, video or audio equipment) whether anyone was present besides the participant(s) and the researcher, and whether the researcher was blind to experimental condition and/or the study hypothesis during data collection.*
Timing	*Indicate the start and stop dates of data collection. If there is a gap between collection periods, state the dates for each sample cohort.*
Data exclusions	*If no data were excluded from the analyses, state so OR if data were excluded, provide the exact number of exclusions and the rationale behind them, indicating whether exclusion criteria were pre-established.*
Non-participation	*State how many participants dropped out/declined participation and the reason(s) given OR provide response rate OR state that no participants dropped out/declined participation.*
Randomization	*If participants were not allocated into experimental groups, state so OR describe how participants were allocated to groups, and if allocation was not random, describe how covariates were controlled.*

Ecological, evolutionary & environmental sciences study design

All studies must disclose on these points even when the disclosure is negative.

Study description	*Briefly describe the study. For quantitative data include treatment factors and interactions, design structure (e.g. factorial, nested, hierarchical), nature and number of experimental units and replicates.*
Research sample	*Describe the research sample (e.g. a group of tagged Passer domesticus, all Stenocereus thurberi within Organ Pipe Cactus National Monument), and provide a rationale for the sample choice. When relevant, describe the organism taxa, source, sex, age range and any manipulations. State what population the sample is meant to represent when applicable. For studies involving existing datasets, describe the data and its source.*
Sampling strategy	*Note the sampling procedure. Describe the statistical methods that were used to predetermine sample size OR if no sample-size calculation was performed, describe how sample sizes were chosen and provide a rationale for why these sample sizes are sufficient.*

Data collection	*Describe the data collection procedure, including who recorded the data and how.*
Timing and spatial scale	*Indicate the start and stop dates of data collection, noting the frequency and periodicity of sampling and providing a rationale for these choices. If there is a gap between collection periods, state the dates for each sample cohort. Specify the spatial scale from which the data are taken*
Data exclusions	*If no data were excluded from the analyses, state so OR if data were excluded, describe the exclusions and the rationale behind them, indicating whether exclusion criteria were pre-established.*
Reproducibility	*Describe the measures taken to verify the reproducibility of experimental findings. For each experiment, note whether any attempts to repeat the experiment failed OR state that all attempts to repeat the experiment were successful.*
Randomization	*Describe how samples/organisms/participants were allocated into groups. If allocation was not random, describe how covariates were controlled. If this is not relevant to your study, explain why.*
Blinding	*Describe the extent of blinding used during data acquisition and analysis. If blinding was not possible, describe why OR explain why blinding was not relevant to your study.*

Did the study involve field work? ☐ Yes ☐ No

Field work, collection and transport

Field conditions	*Describe the study conditions for field work, providing relevant parameters (e.g. temperature, rainfall).*
Location	*State the location of the sampling or experiment, providing relevant parameters (e.g. latitude and longitude, elevation, water depth).*
Access & import/export	*Describe the efforts you have made to access habitats and to collect and import/export your samples in a responsible manner and in compliance with local, national and international laws, noting any permits that were obtained (give the name of the issuing authority, the date of issue, and any identifying information).*
Disturbance	*Describe any disturbance caused by the study and how it was minimized.*

Reporting for specific materials, systems and methods

We require information from authors about some types of materials, experimental systems and methods used in many studies. Here, indicate whether each material, system or method listed is relevant to your study. If you are not sure if a list item applies to your research, read the appropriate section before selecting a response.

Materials & experimental systems

n/a	Involved in the study	
☐	☒	Antibodies
☒	☐	Eukaryotic cell lines
☒	☐	Palaeontology and archaeology
☒	☐	Animals and other organisms
☐	☒	Clinical data
☒	☐	Dual use research of concern
☒	☐	Plants

Methods

n/a	Involved in the study	
☒	☐	ChIP-seq
☒	☐	Flow cytometry
☒	☐	MRI-based neuroimaging

Antibodies

Antibodies used

CyTOF antibodies
Target; Clone; Supplier; Dilution
CD45 HI30 Standard BioTools™ 1:200
CCR6 G034E3 Standard BioTools™ 1:120
CD19 HIB19 Standard BioTools™ 1:100
HLA-DR L243 Standard BioTools™ 1:200
CCR5 NP-6G4 Standard BioTools™ 1:120
CD4 RPA-T4 Standard BioTools™ 1:200
CD8 RPA-T8 Standard BioTools™ 1:200
Tbet 4B10 Biolegend® 1:100
CD16 3G8 Standard BioTools™ 1:200
CD25 2A3 Standard BioTools™ 1:400
OX40 ACT35 Standard BioTools™ 1:200
CD2 TS1/8 Standard BioTools™ 1:400

CCR3 5E8 Biolegend® 1:100
TIM3 F38-2E2 Standard BioTools™ 1:100
CCR10 6588-5 Biolegend® 1:100
CD56 B159 Standard BioTools™ 1:400
PDL-1 29E3.2A3 Standard BioTools™ 1:100
Gata-3 TWAJ eBioscience 1:100
CCR7 G043H7 Standard BioTools™ 1:120
CD28 CD28.2 Standard BioTools™ 1:200
CTLA4 14D3 Standard BioTools™ 1:200
Foxp3 PCH101 Standard BioTools™ 1:133
CXCR3 G025H7 Standard BioTools™ 1:120
CCR4 L291H4 Biolegend® 1:100
CD45RO UCHL1 Standard BioTools™ 1:100
NKG2D ON72 Standard BioTools™ 1:200
CD27 O323 Standard BioTools™ 1:200
RORy 4G419 Thermofisher 1:100
CD45RA HI100 Standard BioTools™ 1:400
CD3 UCHT1 Standard BioTools™ 1:200
Granzyme B GB11 Standard BioTools™ 1:66
Ki67 B56 Standard BioTools™ 1:66
CD137 4B4-1 Standard BioTools™ 1:200
PD-1 EH12.2H7 Standard BioTools™ 1:100
LAG3 11C3C65 Standard BioTools™ 1:100
CD127 A019D5 Standard BioTools™ 1:100
TIGIT MBSA43 Standard BioTools™1:100

Validation

Each antibody has been validated with data available on manufacturer's websites. Antibodies in this panel have also been used and validated in prior publications including in PMID: 34796337, 37904980, and 38260999.

Eukaryotic cell lines

Policy information about cell lines and Sex and Gender in Research

Cell line source(s)

State the source of each cell line used and the sex of all primary cell lines and cells derived from human participants or vertebrate models.

Authentication

Describe the authentication procedures for each cell line used OR declare that none of the cell lines used were authenticated.

Mycoplasma contamination

Confirm that all cell lines tested negative for mycoplasma contamination OR describe the results of the testing for mycoplasma contamination OR declare that the cell lines were not tested for mycoplasma contamination.

Commonly misidentified lines
(See ICLAC register)

Name any commonly misidentified cell lines used in the study and provide a rationale for their use.

Palaeontology and Archaeology

Specimen provenance

Provide provenance information for specimens and describe permits that were obtained for the work (including the name of the issuing authority, the date of issue, and any identifying information). Permits should encompass collection and, where applicable, export.

Specimen deposition

Indicate where the specimens have been deposited to permit free access by other researchers.

Dating methods

If new dates are provided, describe how they were obtained (e.g. collection, storage, sample pretreatment and measurement), where they were obtained (i.e. lab name), the calibration program and the protocol for quality assurance OR state that no new dates are provided.

☐ Tick this box to confirm that the raw and calibrated dates are available in the paper or in Supplementary Information.

Ethics oversight

Identify the organization(s) that approved or provided guidance on the study protocol, OR state that no ethical approval or guidance was required and explain why not.

Note that full information on the approval of the study protocol must also be provided in the manuscript.

Animals and other research organisms

Policy information about studies involving animals; ARRIVE guidelines recommended for reporting animal research, and Sex and Gender in Research

Laboratory animals

For laboratory animals, report species, strain and age OR state that the study did not involve laboratory animals.

Wild animals

Provide details on animals observed in or captured in the field; report species and age where possible. Describe how animals were

Wild animals	*caught and transported and what happened to captive animals after the study (if killed, explain why and describe method; if released, say where and when) OR state that the study did not involve wild animals.*
Reporting on sex	*Indicate if findings apply to only one sex; describe whether sex was considered in study design, methods used for assigning sex. Provide data disaggregated for sex where this information has been collected in the source data as appropriate; provide overall numbers in this Reporting Summary. Please state if this information has not been collected. Report sex-based analyses where performed, justify reasons for lack of sex-based analysis.*
Field-collected samples	*For laboratory work with field-collected samples, describe all relevant parameters such as housing, maintenance, temperature, photoperiod and end-of-experiment protocol OR state that the study did not involve samples collected from the field.*
Ethics oversight	*Identify the organization(s) that approved or provided guidance on the study protocol, OR state that no ethical approval or guidance was required and explain why not.*

Note that full information on the approval of the study protocol must also be provided in the manuscript.

Clinical data

Policy information about clinical studies

All manuscripts should comply with the ICMJE guidelines for publication of clinical research and a completed CONSORT checklist must be included with all submissions.

Clinical trial registration	This was not an interventional clinical trial and is not registered in a publicly accesssible repository.
Study protocol	The study protocol can be requested from the corresponding authors and is not currently publicly available.
Data collection	Patients included in this data analysis were enrolled from May 2021 to October 2022, and data censorship was set at 6 months from last consented patient included in the analysis (April 2023). All patients samples were collected at Johns Hopkins Medicine clinical sites.
Outcomes	Patients were categorized by response vs. non-response based on RECIST v1.1 criteria and documentation by the treating oncologist. IrAEs were defined based on Common Terminology Criteria for Adverse Events version 5 (CTCAE v.5.0). The dates of onset, grade, and duration of irAEs were determined from review by a clinical researcher and confirmed by a medical oncologist reviewer. Progression free survival (PFS) was defined as time from initiation of ICI on study to progression or death, and overall survival (OS) was defined as time from initiation of ICI on study to death. Time to irAE onset was defined as the time from initiation of ICI on study to the onset of the highest grade irAE.

Dual use research of concern

Policy information about dual use research of concern

Hazards

Could the accidental, deliberate or reckless misuse of agents or technologies generated in the work, or the application of information presented in the manuscript, pose a threat to:

No	Yes	
☒	☐	Public health
☒	☐	National security
☒	☐	Crops and/or livestock
☒	☐	Ecosystems
☒	☐	Any other significant area

Experiments of concern

Does the work involve any of these experiments of concern:

No	Yes	
☒	☐	Demonstrate how to render a vaccine ineffective
☒	☐	Confer resistance to therapeutically useful antibiotics or antiviral agents
☒	☐	Enhance the virulence of a pathogen or render a nonpathogen virulent
☒	☐	Increase transmissibility of a pathogen
☒	☐	Alter the host range of a pathogen
☒	☐	Enable evasion of diagnostic/detection modalities
☒	☐	Enable the weaponization of a biological agent or toxin
☒	☐	Any other potentially harmful combination of experiments and agents

Plants

Seed stocks

Report on the source of all seed stocks or other plant material used. If applicable, state the seed stock centre and catalogue number. If plant specimens were collected from the field, describe the collection location, date and sampling procedures.

Novel plant genotypes

Describe the methods by which all novel plant genotypes were produced. This includes those generated by transgenic approaches, gene editing, chemical/radiation-based mutagenesis and hybridization. For transgenic lines, describe the transformation method, the number of independent lines analyzed and the generation upon which experiments were performed. For gene-edited lines, describe the editor used, the endogenous sequence targeted for editing, the targeting guide RNA sequence (if applicable) and how the editor was applied.

Authentication

Describe any authentication procedures for each seed stock used or novel genotype generated. Describe any experiments used to assess the effect of a mutation and, where applicable, how potential secondary effects (e.g. second site T-DNA insertions, mosiacism, off-target gene editing) were examined.

ChIP-seq

Data deposition

- [ ] Confirm that both raw and final processed data have been deposited in a public database such as GEO.
- [ ] Confirm that you have deposited or provided access to graph files (e.g. BED files) for the called peaks.

Data access links
May remain private before publication.

For "Initial submission" or "Revised version" documents, provide reviewer access links. For your "Final submission" document, provide a link to the deposited data.

Files in database submission

Provide a list of all files available in the database submission.

Genome browser session
(e.g. UCSC)

Provide a link to an anonymized genome browser session for "Initial submission" and "Revised version" documents only, to enable peer review. Write "no longer applicable" for "Final submission" documents.

Methodology

Replicates

Describe the experimental replicates, specifying number, type and replicate agreement.

Sequencing depth

Describe the sequencing depth for each experiment, providing the total number of reads, uniquely mapped reads, length of reads and whether they were paired- or single-end.

Antibodies

Describe the antibodies used for the ChIP-seq experiments; as applicable, provide supplier name, catalog number, clone name, and lot number.

Peak calling parameters

Specify the command line program and parameters used for read mapping and peak calling, including the ChIP, control and index files used.

Data quality

Describe the methods used to ensure data quality in full detail, including how many peaks are at FDR 5% and above 5-fold enrichment.

Software

Describe the software used to collect and analyze the ChIP-seq data. For custom code that has been deposited into a community repository, provide accession details.

Flow Cytometry

Plots

Confirm that:

- [ ] The axis labels state the marker and fluorochrome used (e.g. CD4-FITC).
- [ ] The axis scales are clearly visible. Include numbers along axes only for bottom left plot of group (a 'group' is an analysis of identical markers).
- [ ] All plots are contour plots with outliers or pseudocolor plots.
- [ ] A numerical value for number of cells or percentage (with statistics) is provided.

Methodology

Sample preparation

Describe the sample preparation, detailing the biological source of the cells and any tissue processing steps used.

Instrument

Identify the instrument used for data collection, specifying make and model number.

Software

Describe the software used to collect and analyze the flow cytometry data. For custom code that has been deposited into a community repository, provide accession details.

Cell population abundance | *Describe the abundance of the relevant cell populations within post-sort fractions, providing details on the purity of the samples and how it was determined.*

Gating strategy | *Describe the gating strategy used for all relevant experiments, specifying the preliminary FSC/SSC gates of the starting cell population, indicating where boundaries between "positive" and "negative" staining cell populations are defined.*

☐ Tick this box to confirm that a figure exemplifying the gating strategy is provided in the Supplementary Information.

Magnetic resonance imaging

Experimental design

Design type | *Indicate task or resting state; event-related or block design.*

Design specifications | *Specify the number of blocks, trials or experimental units per session and/or subject, and specify the length of each trial or block (if trials are blocked) and interval between trials.*

Behavioral performance measures | *State number and/or type of variables recorded (e.g. correct button press, response time) and what statistics were used to establish that the subjects were performing the task as expected (e.g. mean, range, and/or standard deviation across subjects).*

Acquisition

Imaging type(s) | *Specify: functional, structural, diffusion, perfusion.*

Field strength | *Specify in Tesla*

Sequence & imaging parameters | *Specify the pulse sequence type (gradient echo, spin echo, etc.), imaging type (EPI, spiral, etc.), field of view, matrix size, slice thickness, orientation and TE/TR/flip angle.*

Area of acquisition | *State whether a whole brain scan was used OR define the area of acquisition, describing how the region was determined.*

Diffusion MRI ☐ Used ☐ Not used

Preprocessing

Preprocessing software | *Provide detail on software version and revision number and on specific parameters (model/functions, brain extraction, segmentation, smoothing kernel size, etc.).*

Normalization | *If data were normalized/standardized, describe the approach(es): specify linear or non-linear and define image types used for transformation OR indicate that data were not normalized and explain rationale for lack of normalization.*

Normalization template | *Describe the template used for normalization/transformation, specifying subject space or group standardized space (e.g. original Talairach, MNI305, ICBM152) OR indicate that the data were not normalized.*

Noise and artifact removal | *Describe your procedure(s) for artifact and structured noise removal, specifying motion parameters, tissue signals and physiological signals (heart rate, respiration).*

Volume censoring | *Define your software and/or method and criteria for volume censoring, and state the extent of such censoring.*

Statistical modeling & inference

Model type and settings | *Specify type (mass univariate, multivariate, RSA, predictive, etc.) and describe essential details of the model at the first and second levels (e.g. fixed, random or mixed effects; drift or auto-correlation).*

Effect(s) tested | *Define precise effect in terms of the task or stimulus conditions instead of psychological concepts and indicate whether ANOVA or factorial designs were used.*

Specify type of analysis: ☐ Whole brain ☐ ROI-based ☐ Both

Statistic type for inference (See Eklund et al. 2016) | *Specify voxel-wise or cluster-wise and report all relevant parameters for cluster-wise methods.*

Correction | *Describe the type of correction and how it is obtained for multiple comparisons (e.g. FWE, FDR, permutation or Monte Carlo).*

Models & analysis

n/a	Involved in the study
☐	☐ Functional and/or effective connectivity
☐	☐ Graph analysis
☐	☐ Multivariate modeling or predictive analysis

Functional and/or effective connectivity	*Report the measures of dependence used and the model details (e.g. Pearson correlation, partial correlation, mutual information).*
Graph analysis	*Report the dependent variable and connectivity measure, specifying weighted graph or binarized graph, subject- or group-level, and the global and/or node summaries used (e.g. clustering coefficient, efficiency, etc.).*
Multivariate modeling and predictive analysis	*Specify independent variables, features extraction and dimension reduction, model, training and evaluation metrics.*
